# Is transcranial direct current stimulation beneficial for treating pain, depression, and anxiety symptoms in patients with chronic pain? A systematic review and meta-analysis

**DOI:** 10.3389/fnmol.2022.1056966

**Published:** 2022-12-01

**Authors:** Yu-Rong Wen, Jian Shi, Zheng-Yu Hu, Yang-Yang Lin, You-Tian Lin, Xue Jiang, Rui Wang, Xue-Qiang Wang, Yu-Ling Wang

**Affiliations:** ^1^Department of Sport Rehabilitation, Shanghai University of Sport, Shanghai, China; ^2^College of Kinesiology, Shenyang Sport University, Shenyang, China; ^3^Rehabilitation Medicine Center, The Sixth Affiliated Hospital of Sun Yat-sen University, Guangzhou, China; ^4^Postgraduate Research Institute, Guangzhou Sport University, Guangzhou, China; ^5^Department of Rehabilitation Medicine, Shanghai Shangti Orthopaedic Hospital, Shanghai, China

**Keywords:** transcranial direct current stimulation, chronic pain, depression, anxiety, non-invasive brain stimulation, meta-analysis, systematic review

## Abstract

**Background:**

Chronic pain is often accompanied by emotional dysfunction. Transcranial direct current stimulation (tDCS) has been used for reducing pain, depressive and anxiety symptoms in chronic pain patients, but its therapeutic effect remains unknown.

**Objectives:**

To ascertain the treatment effect of tDCS on pain, depression, and anxiety symptoms of patients suffering from chronic pain, and potential factors that modulate the effectiveness of tDCS.

**Methods:**

Literature search was performed on PubMed, Embase, Web of Science, and Cochrane Library from inception to July 2022. Randomized controlled trials that reported the effects of tDCS on pain and depression and anxiety symptoms in patients with chronic pain were included.

**Results:**

Twenty-two studies were included in this review. Overall pooled results indicated that the use of tDCS can effectively alleviate short-term pain intensity [standard mean difference (SMD): −0.43, 95% confidence interval (CI): −0.75 to −0.12, *P* = 0.007] and depressive symptoms (SMD: −0.31, 95% CI, −0.47 to −0.14, *P* < 0.001), middle-term depressive symptoms (SMD: −0.35, 95% CI: −0.58 to −0.11, *P* = 0.004), long-term depressive symptoms (ES: −0.38, 95% CI: −0.64 to −0.13, *P* = 0.003) and anxiety symptoms (SMD: −0.26, 95% CI: −0.51 to −0.02, *P* = 0.03) compared with the control group.

**Conclusion:**

tDCS may be an effective short-term treatment for the improvement of pain intensity and concomitant depression and anxiety symptoms in chronic pain patients. Stimulation site, stimulation frequency, and type of chronic pain were significant influence factors for the therapeutic effect of tDCS.

**Systematic review registration:**

https://www.crd.york.ac.uk/PROSPERO/display_record.php?RecordID=297693, identifier: CRD42022297693.

## Introduction

Pain is currently defined by the International Association for the Study of Pain as “an unpleasant sensory and emotional experience associated with or resembling that associated with actual or potential tissue damage” (Raja et al., 2020). Pain encompasses sensory, cognitive, and most importantly effective components. As opposed to acute pain, which by definition has <1 month, chronic pain was pain that lasts 3 months or longer (Treede et al., 2019). Chronic pain is a heterogeneous phenomenon caused by multiple pathologies together with chronic somatic tissue degeneration. Different possible strategies for the production of pain may explain various sorts of chronic pain (Ossipov and Porreca, 2007). Under persistent chronic pain, the brain undergoes structural and functional changes, and brain network dynamics are altered (Baliki et al., 2011; Nickel et al., 2012). In adults, the prevalence rate exceeds 50%, and the rate of clinically significant chronic pain is 10–20% (van Hecke et al., 2013). Compared with acute pain, patients with chronic pain are more likely to suffer from dysthymic disorder due to the long course of the disease and its adverse impact on the quality of life of patients. Negative psychological factors, such as depression or anxiety disorders, are usually comorbidities of chronic pain and have morbidity rates of 30–60% and interact to alter disease progression (Walker et al., 2014; Doan et al., 2015). Chronic pain can be a significant risk factor for psychology, and interactively, psychology can exacerbate chronic pain development and disrupt the effectiveness of analgesic therapy. In Europe, 21% of chronic pain patients were diagnosed with depression because of their pain, and most patients with moderate to severe chronic pain do not receive adequate and accurate pain management, which has a serious negative impact on their social work and life (Breivik et al., 2006; van Hecke et al., 2013). Current treatment mainly consists of antidepressants, combined with non-steroidal anti-inflammatory drugs and psychotherapy, etc. Antidepressants and antiepileptics can affect the mental and physical symptoms of depression, pain symptoms of chronic pain, and the overall function of both patients (Reinhold et al., 2011; Bandelow et al., 2012), but only 40–60% of patients have relief from pain and depression and with significant adverse reaction (Zhang and Zhao, 2016). In addition, negative emotions in chronic pain patients were associated with worse opioid outcomes, including decreased pain relief ability and increased likelihood of abuse. These findings underscore the importance of exploring more effective and safer non-pharmacological therapy for pain, anxiety, and depression symptoms in patients with chronic pain.

Transcranial direct current stimulation (tDCS) is a non-invasive brain stimulation method. In clinical practice, tDCS, as a new tool for modulating brain activity (Lefaucheur, 2008), can modulate cortical excitability by delivering a weak constant positive or negative electric current to a target area of the brain *via* electrodes attached to the scalp. Thus, it can directly modulate a wide neural network involved in pain processing through the transcranial application of electrical field stimulation (Antal et al., 2017; O'Connell et al., 2018). In recent years, tDCS has been explored for the treatment of mental and neurological diseases (Meron et al., 2015) such as anxiety and depression (Palm et al., 2016; Vergallito et al., 2021) and has been employed in the treatment of a range of pathological chronic pain circumstances, such as chronic low back pain (CLBP) (Mariano et al., 2019; McPhee and Graven-Nielsen, 2021b), fibromyalgia (Fagerlund et al., 2015; Khedr et al., 2017), and complex regional pain syndrome (Cruccu et al., 2016; Lagueux et al., 2018). However, these results of studies are mixed. The quality of evidence supporting the benefit of tDCS for chronic pains is poor (Knotkova et al., 2021). Notably, to our knowledge, no systematic review has attempted to investigate the effect of tDCS on anxiety and depressive symptoms in patients with chronic pain to gain a more comprehensive understanding of it as a true non-pharmacological therapy for chronic pain. Only a few reviews have focused on the role of tDCS in the treatment of chronic pain in adults but most of these studies either focused on a single chronic pain condition (Mehta et al., 2015; Hou et al., 2016; Alwardat et al., 2020; Lloyd et al., 2020; Yu et al., 2020; Gao et al., 2022) or did not include measures of anxiety and depression in the scopes of systematic review (O'Connell et al., 2011, 2018; Mehta et al., 2015; Alwardat et al., 2020; Lloyd et al., 2020). Therefore, further review and analysis of available evidence on tDCS-related pain, depression, and anxiety in chronic pain patients are necessary. The results of this systematic review and meta-analysis are expected to help clinicians and future researchers provide more sufficient evidence from multiple dimensions to determine the role of tDCS in the treatment of chronic pain and to select ideal tDCS parameters (such as stimulation site, intensity, and duration).

## Methods

### Protocol and registration

This systematic review was reported in line with the PRISMA guidelines (Liberati et al., 2009) and the protocols were prospectively registered on the PROSPERO database with registration number CRD42022297693.

### Search strategy

Our literature search was performed on PubMed, Embase, Web of Science, and Cochrane Library. Publication dates ranged from the first date of availability to July 2022 in all languages. The following keywords were searched: “transcranial direct current stimulation,” “tDCS,” “chronic pain,” “depression,” “depressive syndrome,” “depressive symptom,” and “anxiety.” The complete search strategies are submitted in Supplementary material 1.

### Eligibility criteria

Firstly, studies from four databases were preliminarily selected by their titles and abstracts. If the topic of the article cannot be defined by the title and abstract, we assessed the full text of the article to ascertain whether it could be included in this review. Two evaluators (Y.W. and J.S.) independently assessed studies based on inclusion and exclusion criteria. If the two judges could not reach a consensus, the corresponding author re-evaluated the article and discussed it with them to reach a consensus.

The inclusion criteria were as follows:

- Design of studies: parallel or crossover randomized controlled trials (RCTs)
- Subjects: adults aged more than 18 years old with chronic pain lasting over 3 months
- Types of intervention: transcranial direct current stimulation was used as the main intervention in the experimental group.
- Main outcomes were related to the intensity of pain, and using a validated multi-item scale or structured diagnostic interview for the assessment of depressive symptoms and/or anxiety.

The exclusion criteria were as follows:

- Studies have been published in the form of conference abstracts, dissertations, and books.
- Treatment paradigm did not comply with the published safety guidelines.

### Data extraction

Data extraction for each selected study was completed independently by two evaluators (Y.W. and J.S.) and then reviewed and revised by the corresponding author. If RCTs contained more than two arms, we collected data from the separate treatment arms. A standard information extraction form was jointly designed by two evaluators. Details of data extraction for studies are shown in Supplementary material 2.

### Risk of bias and GRADE

The Cochrane Risk of Bias Tool (Higgins et al., 2019; Sterne et al., 2019) was used by two authors (Y.L. and Z.H.) to independently assess the quality of methods and the risk of bias of these studies. The Cochrane tool categorized the quality risk into three classes: high, low, and unclear which examined potential performance bias, selection bias, attrition bias, detection bias, reporting bias, and other bias.

Furthermore, the quality of evidence for achievements was appraised using the grading of recommendation assessment, development, and evaluation (GRADE) pathway (Atkins et al., 2004). GRADE may reduce the quality of evidence in the systematic evaluation of intervention: inconsistency, risk of bias, inaccuracy, indirectness, and publication bias. GRADEpro was used in evaluating these factors and classifying the quality of evidence into four grades: very low, low, medium, and high quality.

### Data synthesis and analysis

Meta-analysis was executed by employing Stata v16.0 computer program (StataCorp, Texas, USA) with the metan command. The summary effect size (SES) was evaluated by calculating the combined standard mean difference (SMD) of the change score (end-point minus baseline score) and its 95% confidence interval (CI). In the meta-analysis, the SMD was used as a pool-president measure when all studies assessed the same outcome but measured differently (e.g., all studies measured depression but used different psychometric scales). In this case, it was necessary to scale the results to achieve a uniform unit of measure (scale) before combining studies. We interpreted SMD based on previous studies (Varangot-Reille et al., 2022) (0–0.2, trivial; 0.2–0.6, small; 0.6–1.2, moderate; 1.2–2.0, large; 2.0–4.0, very large; >4.0, extremely large). The CI showed the degree to which the true value of this parameter has a certain probability to fall around the measurement result, and it gave credibility to the measured value of the measured parameter. These standardized effect sizes (SES) are separate for the active and sham tDCS interventions. We used the difference obtained by subtracting the baseline values from the short-, medium- and long-term values after the tDCS or sham intervention, respectively, as a comparison of the final SES. At the same time, the heterogeneity was examined using *P*-value and *I*^2^. *P* > 0.1 and *I*^2^ < 50% indicated low heterogeneity, and we selected the fixed effect model. *P* < 0.1 and *I*^2^ > 50% indicated heterogeneity among studies, and the random effect model was adopted. Publication bias was evaluated with a funnel plot. Egger experiment was utilized to appraise whether the degree of asymmetry was significant. Funnel asymmetry due to publication bias was adjusted with the trim and fill method. The reliability of the studies was evaluated through sensitivity assessment, and each investigation was evaluated separately. Three crossover studies were included in this meta-analysis, and these outcomes had no carry-over influences. The generic inverse-variance methods were used to combine the results of the crossover and parallel studies according to Cochrane Handbook for Systematic Reviews of Interventions (Higgins et al., 2019).

A separate meta-analysis was performed to classify the main outcomes into long-term (≥6 weeks post-intervention), medium-term (>1 to 6 weeks post-intervention), and short-term (immediately end of the intervention to ≤1 week post-intervention). In general, when multiple data points were available in different periods, except for short-term results, data closest to the midpoint of the period for extraction was selected. For short-term outcomes, data measured immediately at the end of the intervention had the highest priority.

Three subgroup analyses were set up to investigate factors impacting the result of tDCS on chronic pain with anxiety or depression: type of chronic pain (neuropathic pain vs. non-neuropathic pain vs. visceral pain), stimulation target [M1 vs. dorsolateral prefrontal cortex (DLPFC)], number of sessions (<5 sessions vs. 5–10 sessions vs. >10 sessions).

## Results

### Search results

**Figure 1:**
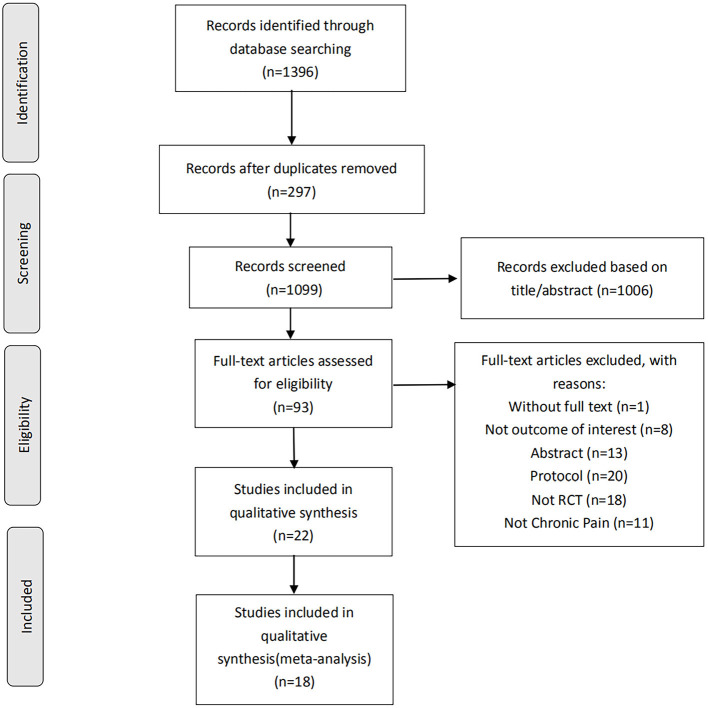
Study selection flowchart according to the PRISMA guidelines, preferred reporting items for systematic reviews and meta-analyses. RCT, randomized controlled trials.

A preliminary search of four databases identified 1,396 articles. In the preliminary search results, 297 duplicate articles were removed, and 1,006 articles with titles and abstracts that did not meet the standard criteria of this review were removed. Next, by evaluating the full text of the excess 93 articles, we subtracted 77 of these studies for several reasons, including without full text (*n* = 1), conference abstracts (*n* = 18), protocol (*n* = 20), not outcome of interest (*n* = 10), not RCT (*n* = 18) and not chronic pain (*n* = 10). The systematic review process is shown in Figure 1. At last, twenty-two RCTs (*n* = 772) met the inclusion criteria, of which eighteen were considered meta-analysis. In all included studies, crossover and parallel investigation designs included 18 parallel investigations and four crossover studies. The fundamental characteristics of all articles are summarized in Table 1.

Table 1Principal characteristics of included studies.

**Table:** 

**References**	**Country**	**Clinical condition, sample size (F/M)**	**Study design**	**Age, mean (years)**	**Outcome measures**	**Baseline pain intensity: mean**	**Baseline depression/anxiety: mean**
Samartin-Veiga et al. (2022)	Spain	Fibromyalgia, 130	RAN-PA-DB-SC	50.37	NRS/HADS	tDCS (M1): 7.43/tDCS (DLPFC): 7 tDCS (OIC): 6.71/sham: 7.49	tDCS (M1) (d): 21.28/tDCS (DLPFC) (d): 21.76 tDCS (OIC) (d): 22.12/sham (d): 20
Forogh et al. (2021)	Iran	Fibromyalgia, 30/0	RAN-PA-SB	45.9	VAS/DASS-21	tDCS: 8.80/rTMS: 7.93	tDCS (d): 25.87/rTMS (d): 23.33 tDCS (a): 22.13/rTMS (a): 16.53
McPhee and Graven-Nielsen (2021a)	Denmark	Low back pain, 18/6	RAN-CO-DB-SC	28.6	VAS/BDI	All: 3.0	All (BDI): 8.7
Gunduz et al. (2021)	USA	Neuropathic pain, 44/68	RAN-PA-DB-SC	44.23	VAS/BDI/BAI	tDCS + MT: 6.18/sham + MT: 6.03	tDCS + MT (d): 8.17/sham + MT (d): 11.68 tDCS + MT (a): 10.51/sham + MT (a): 13.60
Soler et al. (2021)	Spain	Neuropathic pain, 91/39	RAN-PA-SB	48.5	NPSI/BPI/PHQ-9	tDCS: 0.34/sham: 0.31	tDCS (d): 7.05/sham (d): 6.22
Shiasy et al. (2020)	Iran	Low back pain, 60	RAN-PA-SB-SC	32.65	BPI/DASS	ABM + tDCS: 56.13/ABM + sham: 42.86	ABM + tDCS (d): 7.21/ABM + sham (d): 8.61 ABM + tDCS (a): 6.14/ABM + sham (a): 5.69
Young et al. (2020)	Australia	Neuropathic pain, 24/6	RAN-PA-SB-SC	50.53	VAS/DASS	tDCS: 6.3/sham: 5	tDCS (d): 6.9/sham (d): 12.8 tDCS (a): 7.9 /sham (a): 12.1
Dutra et al. (2020)	Brazil	Primary dysmenorrhea, 24/0	RAN-PA-DB-SC	23.55	NRS/HAS	tDCS: 5.07/sham: 4.54	tDCS (a): 19.46/sham (a): 19.45
Pegado et al. (2020)	Brazil	Primary dysmenorrhea, 22/0	RAN-PA-DB-SC	20.82	NRS/HAMA	tDCS: 7.81/sham: 4.54	tDCS (a): 21.9/sham (a): 19.4
Divandari et al. (2019)	Iran	Chronic pelvic pain, 16	RAN-CO-DB-SC	NA	VAS/BDI	tDCS: 6.12/sham: 4.93	tDCS (d): 10.43/sham (d): 8.62
Mariano et al. (2019)	USA	Low back pain, 18/3	RAN-PA-DB-SC	63.08	DVPRS/PASS-20	tDCS: 5.4/sham: 5.5	tDCS (d): 11/sham (d): 8.1 tDCS (a): 34.4/sham (a): 37.5
Yoo et al. (2018)	USA	Fibromyalgia, 3/55	RAN-PA-SB-SC	46.53	NRS/BDI	tDCS: 6.75/sham: 7.19	tDCS (d): 21.75/sham (d): 19.9
Ibrahim et al. (2018)	Egypt	Visceral pain, 27/13	RAN-PA-DB-SC	57.87	VAS/HAMD	tDCS: 6.5/sham: 6.85	tDCS (d): 17/sham (d): 16.85
Morin et al. (2017)	Canada	Vestibulodynia, 39	RAN-PA-DB-SC	22	NRS/BDI/PASS-20	tDCS: 6.5/sham: 7	tDCS (d): 7.1/sham (d): 6.2 tDCS (a): 42.7/sham (a): 33.9
Mendonca et al. (2016)	Brazil	Fibromyalgia, 44/1	RAN-PA-DB-SC	47.6	VNS/BDI	tDCS + AE: 7.3/sham + AE: 6.8	tDCS + AE (d): 20.8/sham + AE (d): 21.0
Ayache et al. (2016)	France	Neuropathic pain, 13/3	RAN-CO-DB-SC	48.9	VAS/HADS	tDCS: 51.2/sham: 52.1	tDCS (d): 6.4/Sham (d): 6.3 tDCS (a): 7.7 /Sham (a): 8.1
Fagerlund et al. (2015)	Norway	Fibromyalgia, 45/3	RAN-PA-DB-SC	48.6	NRS/HADS	tDCS: 4.93/sham: 5.31	tDCS (d): 4.94/sham (d): 5.71 tDCS (a): 7.00/sham (a): 5.76
Kim et al. (2013)	Korea	Neuropathic pain, 35/25	RAN-PA-DB-SC	61.57	VAS/BDI	tDCS (M1): 5.75/tDCS (DLPFC): 5.70/sham: 5.55	tDCS (M1) (d): 10.60/tDCS (DLPFC) (d): 8.75/sham (d): 11.10
Wrigley et al. (2013)	Australia	Neuropathic pain, 2/8	RAN-CO-DB-SC	56.1	NRS/BDI	All: 5.6	All (d): 8.4
Mori et al. (2010)	Italy	Neuropathic pain, 11/8	RAN-PA-DB-SC	44.8	VAS/BDI	tDCS: 55.5/sham: 57.7	tDCS (d): 11.1/Sham (d): 8.79 tDCS (a): 37.7/Sham (a): 38.7
Fregni et al. (2006a)	USA	Neuropathic pain, 14/3	RAN-PA-DB-SC	35.75	VAS/BDI	tDCS: 6.2/sham: 6	tDCS (d): 8.9/sham (d): 12.6
Fregni et al. (2006b)	USA	Fibromyalgia, 32/0	RAN-PA-DB-SC	52.73	VAS/BDI	tDCS (M1): 8.5/tDCS (DLPFC): 8/sham : 7.5	tDCS (M1) (d): 19.9/tDCS (DLPFC) (d): 17.8/sham (d): 20.7

M, Male; F, Female; d, Depression; a, Anxiety; VAS, Visual Analogue Scale; NRS, Numeric Rating Scale; BPI, Brief Pain Inventory; DVPRS: Defense and Veterans Pain Rating Scale; VNS, Visual Numeric Scale; NPSI, Neuropathic Pain Symptom Inventory; BDI, Beck depression inventory; DASS-21, Depression Anxiety Stress Scale-21; HAM-D, Hamilton rating scale for depression; HAM-A, Hamilton rating scale for anxiety; HAS, Hamilton Anxiety Scale; PHQ-9: Patient Health Questionnaire 9-item; PASS-20, Pain Anxiety Symptoms Scale; HADS, Hospital Anxiety and Depression Scale; RAN, randomized; CO, cross-over; PA, parallel; DB, double-blind; SB, single-blind; SC, sham-controlled; NA, not available.

**Table:** 

**References**	**Mode**	**Site**	**Intensity**	**Duration**	**Intervention/control**	**Adverse effects**
Samartin-Veiga et al. (2022)	Anodal	M1/DLPFC/OIC	2 mA	20 min/15 sessions	tDCS/sham tDCS	Tickling/itching
Forogh et al. (2021)	Anodal	DLPFC	2 mA	20 min/3 sessions	tDCS/rTMS	NA
McPhee and Graven-Nielsen (2021a)	Anodal	mPFC	2 mA	20 min/3 sessions	tDCS/sham tDCS	Skin redness/headache/nausea fatigue/insomnia/sleepiness
Gunduz et al. (2021)	Anodal	M1	2 mA	20 min/10 sessions	MT + tDCS/MT + sham tDCS	Sleepiness/neck pain/tingling/headache/scalp pain/acute mood change/skin redness
Soler et al. (2021)	Anodal	M1	2 mA	20 min/10 sessions	VI + tDCS/VI + sham tDCS	Tingling
Shiasy et al. (2020)	Anodal	M1	2 mA	20 min/5 sessions	ABM + tDCS/ABM + sham tDCS	NA
Young et al. (2020)	Anodal	M1	2 mA	20 min/5 sessions	tDCS/sham tDCS	NA
Dutra et al. (2020)	Anodal	DLPFC	2 mA	20 min/5 sessions	tDCS/sham tDCS	Tingling
Pegado et al. (2020)	Anodal	M1	2 mA	20 min/5 sessions	tDCS/sham tDCS	Tingling
Divandari et al. (2019)	Anodal	M1/DLPFC	0.3 mA	20 min/1 session	tDCS/sham tDCS	NO
Mariano et al. (2019)	Cathodal	dACC	2 mA	20 min/10 sessions	tDCS/sham tDCS	NA
Yoo et al. (2018)	Cathodal/anodal	DLPFC	2 mA	20 min/5 sessions	tDCS/sham tDCS	NA
Ibrahim et al. (2018)	Anodal	M1	2 mA	30 min/10 sessions	tDCS/sham tDCS	NO
Morin et al. (2017)	Anodal	M1	2 mA	20 min/10 sessions	tDCS/sham tDCS	Tingling/skin redness
Mendonca et al. (2016)	Anodal	M1	2 mA	20 min/5 sessions	AE + tDCS/ AE + sham tDCS	Headache/neck pain/tingling/skin redness
Ayache et al. (2016)	Anodal	DLPFC	2 mA	20 min/3 sessions	tDCS/sham tDCS	Insomnia/nausea/headache
Fagerlund et al. (2015)	Anodal	M1	2 mA	20 min/5 sessions	tDCS/sham tDCS	Headache/neck pain/scalp pain/Tingling/Itching
Kim et al. (2013)	Anodal	M1/DLPFC	2 mA	20 min/5 sessions	tDCS/sham tDCS	Tingling/fatigue/itching/headache/insomnia
Wrigley et al. (2013)	Anodal	M1	2 mA	20 min/5 sessions	tDCS/sham tDCS	Tingling/headache/fatigue/nausea/skin redness
Mori et al. (2010)	Anodal	M1	2 mA	20 min/5 sessions	tDCS/sham tDCS	No
Fregni et al. (2006a)	Anodal	M1	2 mA	20 min/5 sessions	tDCS/sham tDCS	Headache/itching
Fregni et al. (2006b)	Anodal	M1/DLPFC	2 mA	20 min/5 sessions	tDCS/sham tDCS	Sleepiness/headache

DLPFC, dorsolateral prefrontal cortex; M1, primary motor cortex; mPFC, medial prefrontal cortex; OIC, operculo-insular cortex; dACC, dorsal anterior cingulate cortex; NA, not available; VI, visual illusion; MT, mirror therapy; AE, aerobic exercise; ABM, attention bias modification.

### Characteristics of the included studies

The considered investigations were published from 2006 to 2022. In all tDCS studies, the sample size ranged from 10 to 130. Studies covered a wide variety of pain types. Most trials enrolled male and female patients except (Fregni et al., 2006b; Forogh et al., 2021) (fibromyalgia); (Divandari et al., 2019) (chronic pelvic pain); (Morin et al., 2017) (provoked vestibulodynia); and (Dutra et al., 2020; Pegado et al., 2020) (chronic abdominal pain), which recruited females only. One study did not present information on gender distribution (Samartin-Veiga et al., 2022). Almost all the included RCTs evaluated chronic pain intensity with self-reported scales (VAS or NRS). We investigated outcomes that unmistakably presented the scores of self-announced depression or anxiety. Detailed descriptions are placed in Supplementary material 3.

### Short-term (0–1 week post-intervention)

#### Pain intensity

**Figure 2:**
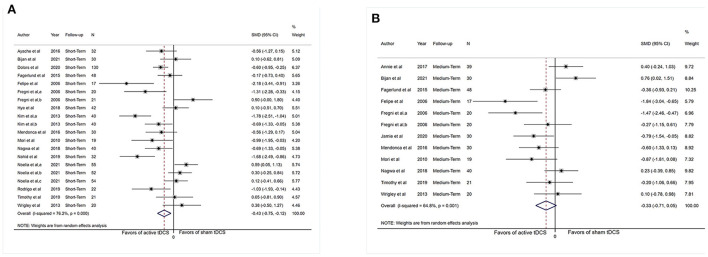
Forest plot showing overall effect sizes (Hedges' g) of real tDCS on pain intensity within studies. These plots show the pooled SMD (large diamond shape) and *I*^2^ resulting from the meta-analysis. **(A)** Pain scores from short-term data. **(B)** Pain scores from medium-term data. CI, confidence interval; SMD, standard mean difference; tDCS, transcranial direct current stimulation; a, motor cortex; b, dorsolateral prefrontal cortex; c, operculo-insular cortex.

Sufficient information was accessible from 16 investigations (*n* = 683) for short-term assessment. We did not extract data from (Morin et al., 2017; Young et al., 2020) as the necessary chronic pain scores were not available for the short-term analysis. Figure 2A revealed an overall effect of real stimulation (SMD: −0.43, 95% CI: −0.75 to −0.12, *P* = 0.007), but with considerable heterogeneity (*I*^2^ = 76.2%, *P* < 0.001).

**Figure 3:**
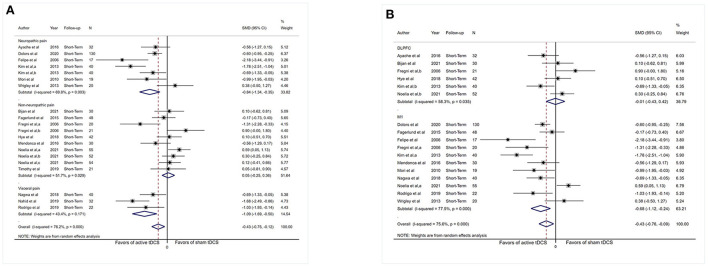
Forest plot showing SMDs in pain intensity scores from data of short-term studies. **(A)** Sub-analysis of pain intensity scores by different types of chronic pain patients. **(B)** Sub-analysis of pain intensity scores comparing the stimulation of M1 with DLPFC. CI, confidence interval; SMD, standard mean difference; tDCS, transcranial direct current stimulation; M1, motor cortex; DLPFC, dorsolateral prefrontal cortex; a, motor cortex; b, dorsolateral prefrontal cortex; c, operculo-insular cortex.

Subgrouping analysis by painful conditions significantly increased the effect size and decreased heterogeneity in the visceral pain subgroup (SMD: −1.09, 95% CI: −1.69 to −0.50, *P* < 0.001, *I*^2^ = 43.4%, *P* = 0.17) and neuropathic pain subgroup (SMD: −0.84, 95% CI: −1.34 to −0.35, *P* = 0.001, *I*^2^ = 69.8%, *P* = 0.003) but did not show effect size in the non-neuropathic pain subgroup (SMD: 0.05, 95% CI: −0.25 to 0.36, *P* = 0.72, *I*^2^ = 51.7%, *P* = 0.003; Figure 3A).

Analysis limited to comparisons of the M1 stimulation (*n* = 441) did not reduced heterogeneity substantially (*I*^2^ = 77.5%, *P* < 0.001) and displayed an effect (SMD: −0.68, 95% CI: −1.12 to −0.24, *P* = 0.003). Analysis limited to comparisons of DLPFC stimulation (*n* = 217) reduce heterogeneity (*I*^2^ = 58.3%, *P* = 0.035), and no evidence of an influence of DLPFC tDCS on pain severity was obtained (SMD: −0.01, 95% CI: −0.43 to 0.42, *P* = 0.98; Figure 3B).

Studies were categorized by the number of sessions (<5 sessions vs. 5–10 sessions vs. >10 sessions). In real tDCS group, no evidence of an effect of less than five sessions of tDCS stimulation (SMD: −0.70, 95% CI: −1.68 to 0.28, *P* = 0.16) and more than 10 sessions of tDCS stimulation (SMD: 0.33, 95% CI: 0.02–0.65, *P* = 0.04) for pain intensity was obtained. Five to ten sessions of tDCS stimulation revealed an effect (SMD: −0.57, 95% CI: −0.92 to −0.21, *P* = 0.002). Overall, considerable heterogeneity was observed (see Supplementary Figure S1).

#### Depression

**Figure 4:**
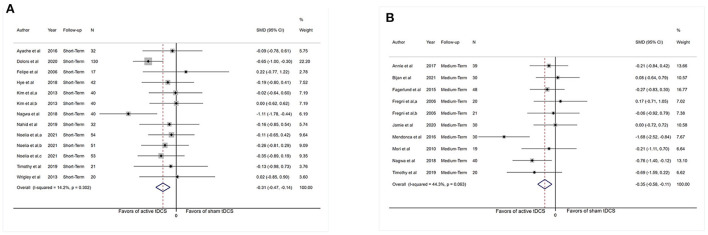
Forest plot showing overall effect sizes (Hedges' g) of real tDCS on depression within studies. These plots show the pooled SMD (large diamond shape) and *I*^2^ resulting from the meta-analysis. **(A)** Depression scores from short-term data. **(B)** Depression scores from medium-term data. CI, confidence interval; SMD, standard mean difference; tDCS, transcranial direct current stimulation; a, motor cortex; b, dorsolateral prefrontal cortex; c, operculo-insular cortex.

Adequate short-term information was available from 10 studies for depression analysis (*n* = 502). The pooled SMD for the aforesaid finding was −0.31 (95% CI: −0.47 to −0.14, *P* < 0.001; Figure 4A).

#### Anxiety

**Figure 5:**
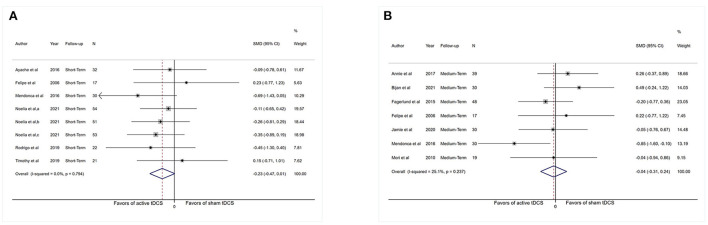
Forest plot showing overall effect sizes (Hedges' g) of real tDCS on anxiety within studies. These plots show the pooled SMD (large diamond shape) and *I*^2^ resulting from the meta-analysis. **(A)** Anxiety scores from short-term data. **(B)** Anxiety scores from medium-term data. CI, confidence interval; SMD, standard mean difference; tDCS, transcranial direct current stimulation; a, motor cortex; b, dorsolateral prefrontal cortex; c, operculo-insular cortex.

Adequate short-term information was available from six studies for anxiety analysis (*n* = 230). Figure 5A shows no apparent reduction in anxiety scores (SMD: −0.23, 95% CI: −0.47 to 0.01, *P* = 0.06). The *I*^2^-test revealed heterogeneity of 0% (*P* = 0.79). More medium-term (1–6 weeks post-intervention) and long-term (>6 weeks post-intervention) results were presented in Supplementary material 3.

### Medium-term (1–6 weeks post-intervention)

#### Pain intensity

Sufficient data were accessible from 11 studies (*n* = 324) for medium-term analysis. There was heterogeneity (*I*^2^ = 64.8%, *P* = 0.001) and Figure 2B revealed no significant reduction in pain intensity in active tDCS stimulation (SMD: −0.33, 95% CI: −0.71 to 0.05, *P* = 0.09).

**Figure 6:**
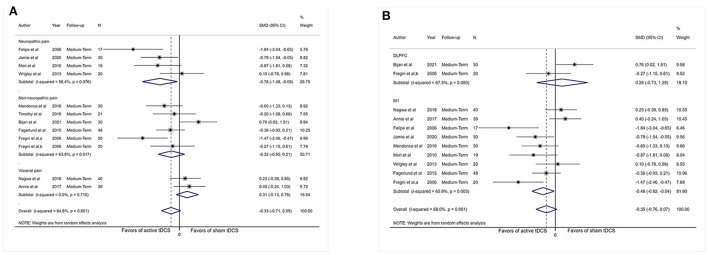
Forest plot showing SMDs in pain intensity scores from data of medium-term studies. **(A)** Sub-analysis of pain intensity scores by different types of chronic pain patients. **(B)** Sub-analysis of pain intensity scores comparing the stimulation of M1 with DLPFC. CI, confidence interval; SMD, standard mean difference; tDCS, transcranial direct current stimulation; M1, motor cortex; DLPFC, dorsolateral prefrontal cortex; a, motor cortex; b, dorsolateral prefrontal cortex.

Subgrouping studies by type of painful condition significantly diminished heterogeneity in the visceral pain subset (*I*^2^ = 0%, *P* = 0.71). Pooling data from these studies in neuropathic pain subgroup showed an effect in favor of the real intervention (SMD: −0.78, 95% CI: −1.48 to −0.08, *P* = 0.03; Figure 6A). No effect of pain intensity reduction was observed in the visceral pain subgroup (*P* = 0.17) and non-neuropathic pain subgroup (*P* = 0.24).

Assessment confined to comparisons of M1 stimulation (*n* = 263) did not reduce heterogeneity dramatically (*I*^2^ = 65.9%, *P* = 0.003) and revealed an influence (SMD: −0.48, 95% CI: −0.92 to −0.04, *P* = 0.03). Analysis was confined to comparisons of DLPFC stimulation (*n* = 50), and no indication of effect of DLPFC tDCS for analgesic effect was observed (SMD: 0.28, 95% CI: −0.73–1.28, *P* = 0.60; Figure 6B), and substantial heterogeneity was demonstrated (*I*^2^ = 67.5%, *P* = 0.08).

Studies were categorized by the number of sessions. In this group, no indication of an effect of fewer than five sessions of tDCS stimulation (SMD: 0.76, 95% CI: 0.02–1.51, *P* = 0.04) for pain intensity was obtained. Five to ten sessions of tDCS stimulation demonstrated an effect (SMD: −0.43, 95% CI: −0.76 to −0.06, *P* = 0.02), and low heterogeneity was observed (*I*^2^ = 37.9%, *P* = 0.13; Supplementary Figure S1).

#### Depression

Adequate medium-term data were accessible from nine studies (*n* = 287) for depression analysis. The pooled SMD for this outcome was −0.35 (95% CI: −0.58 to −0.11, *P* = 0.004). The *I*^2^-test revealed heterogeneity of 44.3% (*P* = 0.10; Figure 4B).

#### Anxiety

Adequate medium-term data were accessible from seven studies (*n* = 213) for anxiety analysis. Meta-analysis showed no substantial reduction in anxiety scores (SMD: −0.04, 95% CI: −0.31–0.24, *P* = 0.79). The *I*^2^-test revealed heterogeneity of 25.1% (*P* = 0.24; Figure 5B).

### Long-term (>6 weeks post-intervention)

#### Pain intensity

Three investigations (*n* = 92) provided long-term data for pain analysis (Mendonca et al., 2016; Morin et al., 2017; Forogh et al., 2021). High heterogeneity was observed (*I*^2^ = 71.9%, *P* = 0.03), and no influence for real tDCS was presented (SMD: 0.46, 95% CI: −0.35–1.27, *P* = 0.27; Supplementary Figure S2).

#### Depression

Adequate long-term data (*n* = 193) were available from four studies (Mendonca et al., 2016; Morin et al., 2017; Forogh et al., 2021; Samartin-Veiga et al., 2022) for depression analysis. The pooled SMD for this outcome was −0.38 (95% CI: −0.64 to −0.13, *P* = 0.003). The *I*^2^-test revealed heterogeneity of 38.4% (*P* = 0.15; Supplementary Figure S3).

#### Anxiety

Adequate long-term data (*n* = 213) were accessible from five studies for anxiety analysis. The pooled SMD for this comparison was −0.26 (95% CI: −0.51 to −0.02, *P* = 0.04). The *I*^2^-test revealed no heterogeneity of 28.8% (*P* = 0.21; Supplementary Figure S4).

### Risk of bias and GRADE

**Figure 7:**
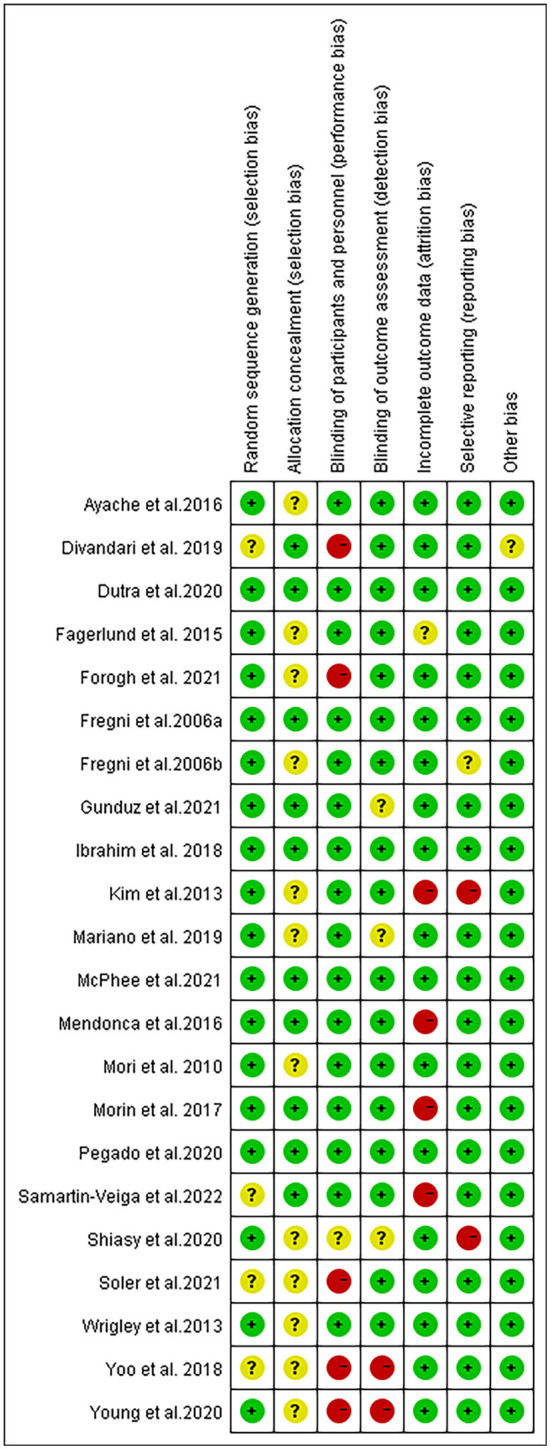
Methodological quality summary: review authors' judgements about each methodological quality item for each included study.

**Figure 8:**
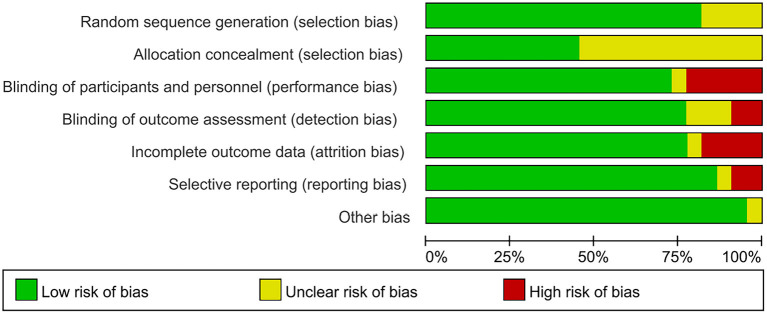
Risk of bias graph: review authors' judgements about each risk of bias item presented as percentages across all included studies.

A brief description of the risk of bias evaluation for each investigation is presented in Figures 7, 8 and Supplementary material 3. Overall, 10 studies found substantial risk of bias across four of the seven criteria (Kim et al., 2013; Mendonca et al., 2016; Morin et al., 2017; Yoo et al., 2018; Divandari et al., 2019; Shiasy et al., 2020; Young et al., 2020; Forogh et al., 2021; Soler et al., 2021; Samartin-Veiga et al., 2022). The quality of evidence assessed by the GRADE approach is shown in Supplementary material 4.

### Sensitivity analysis and publication bias

Removing the literature comparisons one by one did not significantly change the heterogeneity of the pain outcomes. No outliers were found because the total effect size of each investigation was within 2 S.D. of the total average impact size. Additional meta-analysis was conducted to determine whether the exclusion of two high-risk biased trials influences the outcome of a short-term meta-analysis. The other 14 studies maintained statistically meaningful impact estimate of −0.37 (95% CI: −0.70 to −0.04) with heterogeneity of 74.1% (Ayache et al., 2016). The results of publication bias were placed in Supplementary material 3.

### Adverse event of intervention

Among the studies, there were 14 minor adverse reactions reported after the intervention. including tingling, itching, and skin redness under the area of stimulation (Fregni et al., 2006a; Kim et al., 2013; Wrigley et al., 2013; Fagerlund et al., 2015; Mendonca et al., 2016; Morin et al., 2017; Dutra et al., 2020; Pegado et al., 2020; Gunduz et al., 2021; McPhee and Graven-Nielsen, 2021a; Soler et al., 2021; Samartin-Veiga et al., 2022); headache (Fregni et al., 2006a,b; Kim et al., 2013; Wrigley et al., 2013; Fagerlund et al., 2015; Ayache et al., 2016; Mendonca et al., 2016; Gunduz et al., 2021; McPhee and Graven-Nielsen, 2021a); nausea (Wrigley et al., 2013; Ayache et al., 2016; McPhee and Graven-Nielsen, 2021a); fatigue (Kim et al., 2013; Wrigley et al., 2013; McPhee and Graven-Nielsen, 2021a); insomnia (Kim et al., 2013; Ayache et al., 2016; McPhee and Graven-Nielsen, 2021a); neck pain (Fagerlund et al., 2015; Mendonca et al., 2016; Gunduz et al., 2021); sleepiness (Fregni et al., 2006b; Gunduz et al., 2021; McPhee and Graven-Nielsen, 2021a); acute mood change (Gunduz et al., 2021); and scalp pain (Fagerlund et al., 2015; Gunduz et al., 2021). However, they were evenly distributed among the real and sham stimulus groups. Three studies showed that no patients experienced adverse effects after the stimulation (Mori et al., 2010; Ibrahim et al., 2018; Divandari et al., 2019) and it was not mentioned in five of the studies (Yoo et al., 2018; Mariano et al., 2019; Shiasy et al., 2020; Young et al., 2020; Forogh et al., 2021).

## Discussion

### Main findings

In previous studies, a large number of studies on tDCS have, respectively, affirmed its efficacy in chronic pain and mood disorders (especially depression), but we know little about the efficacy of tDCS in the treatment of both symptoms. This is the first systematic review (including 18 studies) on the effects of tDCS on chronic pain, depressive and anxiety symptoms.

Low quality of evidence did show a substantial reduction in pain intensity in comparison with the sham intervention at 0–1 week. However, the results showed heterogeneity. Evidence from medium- and long-term follow-ups did not suggest that tDCS is effective after 1 week. The tDCS showed statistical significance for pain relief in the short-term but no effect was shown in long-term follow-up, a finding consistent with the previous study (O'Connell et al., 2018). O'Connell et al. found that active tDCS is superior to the method used in the control group in the total sample (SMD: −0.43, 95% CI: −0.63 to −0.22, *P* < 0.001) in the short-term stage, the difference is a 17% alteration in chronic pain, which reached the threshold of clinically essential discrepancy. But no effect was observed in long-term follow-up. Medium-term data showed that the findings of this review and O'Connell et al.'s study contradict each other. The largest factor contributing to this difference may come from the type of subjects included. O'Connell included patients with only chronic pain. By contrast, we included people with chronic pain and dysthymic disorder. It could be seen that depression or anxiety mediates the effect of tDCS on chronic pain.

Most tDCS trials have specifically recruited participants who have not responded to current clinical treatments for pain relief. Therefore, we recognize that this analysis largely reflects the importance of the efficacy of tDCS for refractory chronic pain, but may not accurately reflect their efficacy for all chronic pain. In addition, we find that the short-term influences detected for tDCS on chronic pain with psychological disorders might be overstated through the advantage of small-scale research benefits, heterogeneity of design, uncertain risk of bias, and restrictions of investigation approaches. In long-term data, the incentive to analyze effect sizes also appeared to be insufficient due to the reduced number of studies. Future research may have a substantial impact on the evaluation of the efficacy presented.

For depression improvement in patients with chronic pain, moderate quality evidence suggested that tDCS stimulation had a significant and sustained effect. This result complemented the previous research conclusions of tDCS in a clean sample of patients suffering from depression (Moffa et al., 2020; Zhang et al., 2021), which proves that tDCS still has the effect of improving depression in chronic pain patients. The pooled analysis results from the short to medium term did not show any effect of real tDCS on anxiety. Very poor-quality evidence suggested that tDCS can have long-term influences on anxiety.

Pooled data from all of our studies found that tDCS stimulation of M1 and DLPFC had completely different effect sizes in reducing pain, depression, and anxiety. In the studies done before, DLPFC and M1 were the most regular sites of stimulation, among which M1 was the most abundant. Of the 18 quantitative analyses in our study, 13 studies stimulated M1. Positive stimulation of M1 was directly associated with analgesia and relief of depression. However, the stimulation of DLPFC showed no effect. Four out of six studies (Fregni et al., 2006b; Yoo et al., 2018; Forogh et al., 2021; Samartin-Veiga et al., 2022) that stimulated the DLPFC in tDCS articles had no apparent effect compared to the control group. Fregni et al. (2006b) executed a three-arm investigation comparing the influences of M1 stimulation, DLPFC stimulation, and sham stimulation in cases suffering from fibromyalgia. They found that only anodic stimulation at M1 still had significant analgesic effects after 3 weeks of follow-up, but not at DLPFC.

Our subgroup analysis found that tDCS has a greater therapeutic effect on neuropathic pain but showed no vital enhancements in non-neuropathic and visceral pain. Our study did not find any serious complications of tDCS, and it was well-tolerated.

### Effect of tDCS on pain intensity, depression, and anxiety

In this review, chronic pain with depression or anxiety was the focus. Chronic pain, anxiety, and depression can occur together. According to the effective application of tricyclic antidepressants in chronic neuropathic pain, modern research theories propose that chronic pain has a common physiological and pathological mechanism with depression and anxiety (Sacks et al., 2018).

Notably, regulating depression and anxiety is an important reason for the improvement of chronic pain (Marshall et al., 2017). Many studies have shown that tDCS stimulation improves patients' depression symptoms and reduces pain intensity (Lorenz et al., 2003; Khedr et al., 2017; Hassan et al., 2021). Given the connection between pain, depression, and anxiety, we could expect that reduced depression or anxiety may decrease pain. However, the current study did not fully approve of this hypothesis. The improvement of anxiety and analgesia effect is not consistent. Improvements in depression were consistent with improvements in pain intensity in the short term, but not in medium and long-term follow-up. The changes in anxiety and pain intensity were completely discordant. We speculate that depression may have a greater effect on chronic pain. Nevertheless, this finding was difficult to interpret at present and may be related to the complex mechanisms underlying the comorbidity of pain and mood disorders and the uncertainty of long-term follow-up.

Based on previous research findings, the mechanisms of chronic pain comorbidities with emotional disorders may include the following two systems: central nervous system regulation mechanism and endocrine regulation mechanism. The neuromodulatory system includes the midbrain, hypothalamus, peripheral cortex, and brain stem. It participates in emotional and pain regulation activities, thus triggering the co-occurrence of pain and psychological disorder. The view has also been proved by neuroimaging studies which demonstrated the anterior cingulate cortex, prefrontal cortex, nucleus accumbens and amygdala are overlapping brain regions of chronic pain and depression. Meanwhile, neuroimaging results also showed that the shared mechanism of dysregulation between emotion and reward process has something in common (Tappe-Theodor and Kuner, 2019). The endocrine regulatory system is dominated by hypothalamic–pituitary–adrenocortical (HPA) system, and stress response leads to the disorder of endocrine system, which in turn leads to pain and depression. Therefore, the potential mechanisms may be associated with the HPA axis in stress and chronic pain. Notably, chronic pain is positively correlated with widespread anxiety because it is a common condition coexisting with anxiety (Bair et al., 2008; Mundal et al., 2014). In fact, individuals have high baseline anxiety before experiencing chronic pain onset (Gupta et al., 2007). Studies on the relationship between anxiety and chronic pain are much fewer than those on depression and chronic pain. Thus, the role of anxiety in pain may be far ignored. The mechanism between chronic pain and anxiety, like depression, is full of complex factors (Edwards et al., 2006; Campbell and Edwards, 2009; Niederstrasser et al., 2014) which may involve pro-inflammatory immune responses, stress, indices of central sensitization, central nociceptive processing system. However, our confidence in this conclusion may be limited by the small subject numbers. The improvement of anxiety may need a long-term process, because the effectiveness of tDCS on anxiety in chronic pain patients seems to be reflected in follow-up after 6 weeks. In addition, the improvement of anxiety and pain outcomes may be affected by gender. There was research data (Harvie et al., 2021) supported that the relationships between some anxiety and chronic pain are moderated by sex. Thus, subgrouping by status and sex in future studies is recommended to explore psychological factors of chronic pain among individuals.

### Target of stimulation

According to the results of this meta-analysis, different target areas stimulated by tDCS could significantly affect pain and emotional outcomes. More and more evidence suggested that there may be different mechanisms for the analgesic effect of tDCS stimulation of M1 and DLPFC (Mhalla et al., 2011). The effects of M1 tDCS were thought to alter pain sensory discrimination by restraining lateral thalamic activity, which may change the function and connection of the thalamus and hypothalamic nucleus through motor disinhibition, and regulate the emotional components of pain. One study demonstrated that ten times of M1-targeted anodal tDCS stimulation can effectively relieve pain severity and depressive disorder in fibromyalgia (Mhalla et al., 2011), similar to the report of Kang et al. (2020). This finding may be related to the relationship between M1 and the thalamus, and the somatosensory cortex can be controlled directly through the cortico-cortical M1-S1 pathways (DosSantos et al., 2016).

A clinical guideline (Fregni et al., 2021) based on a systematic review and meta-analysis pointed out that anodal tDCS placed in L-DLPFC is indeed effective in improving depressive disorder and was listed as a class A recommendation. In clinical research of repetitive transcranial magnetic stimulation in the therapy of refractory depression, O'Reardon et al. (2007) demonstrated that L-DLPFC stimulation demonstrated a favorable effect on pain relief unexpectedly. PFC is an area directly involved in cognitive pain interpretation (Seminowicz and Moayedi, 2017). The L-DLPFC stimulation in healthy participants showed that tDCS can induce increased perfusion in some parts of brain regions, including the insular cortex, cingulate cortex, and periaqueductal gray (Stagg et al., 2013). DLPFC combined with limbic system can regulate the perception of pain. Pain inhibition through descending fibers of the prefrontal cortex as a top-down mechanism has been proposed. In general, tDCS may affect multiple systems, the tonic control of pain through the activity of cortical thalamic pathway or DMN and the emotional control of pain through marginal connection (Keeser et al., 2011; Kucyi et al., 2013; Clarke et al., 2014; Egorova et al., 2015; Flood et al., 2016). A bi-directional association was observed between psychological disorders and chronic pain (Kroenke et al., 2011). Therefore, the tDCS stimulation of DLPFC has great potential for analgesia. Although owing to the small sample size and lack of evidence, recommendations about the stimulation of tDCS in the DLPFC are inconclusive.

### Types of pain

Benefits for neuropathic pain have conflicting evidence. Our subgroup analysis found that tDCS has a greater therapeutic effect on neuropathic pain but showed no vital enhancements in non-neuropathic and visceral pain. These results were consistent with previous RCTs showing that tDCS has little or no effect on analgesia, particularly fibromyalgia (Luedtke et al., 2012; Zhu et al., 2017), chronic pelvic pain and CLBP (Alwardat et al., 2020). In a recent meta-analysis that included five investigations of tDCS for chronic non-specific low back pain, the results revealed multiple sessions of tDCS were not statistically better than sham intervention (Alwardat et al., 2020). Leung et al. (2009) noted that tDCS had a greater influence on central pain compared to peripheral neuropathic pain however this was not statistically significant. Nevertheless, O'Connell's review of chronic pain concluded that active stimulation had analgesic effects in non-neuropathic pain but not in neuropathic pain which contradicts our findings. This difference may be due to the different kinds of chronic pain that the patients included in the study suffered. Chronic pain is a heterogenous phenomenon that results from a wide variety of pathologies. Dysthymic disorders may mediate the therapeutic effects of tDCS. It is likely that different mechanisms of pain production underpin these different effects of chronic pain.

### Dose-effect response

The analgesic effects of tDCS stimulation may vary from multiple sessions (Cruccu et al., 2016; O'Connell et al., 2018). The tDCS impacts are cumulative, and multiple sessions are purportedly required to achieve clinically useful results (Woods et al., 2016). The minimum and maximum effects of tDCS have not been fully studied. In the present analysis, tDCS stimulation with 5 to 10 sessions seems to show effectiveness in reducing pain in patients with mood disorders, which showed that the duration of treatment for tDCS to produce maximum effect is not as long as possible. McPhee and Graven-Nielsen (2021a) applied 2 mA, three sessions of HD-tDCS stimulation to mPFC targets in CLBP patients. Active HD-tDCS showed no significant effects on anti-nociceptive mechanisms, nor on other psychophysical tests, clinical LBP features, or psychological characteristics. Samartin-Veiga et al. (2022) applied 2 mA, 15 sessions of tDCS stimulation to M1, DLPFC, and operculo-insular cortex in fibromyalgia patients, The results did not show that the analgesic effect of real tDCS was superior to that of the sham control group. In addition, we found that few studies selected tDCS stimulation with more than 10 sessions in cases suffering from chronic pain comorbidities possibly because of the difficulty and complexity of the study design. According to studies of patients with severe depression, the duration of its effects is long. Future studies should focus more on long sessions (15–20) to further determine the positive effects of tDCS on pain improvement following the end of the session and in a long follow-up period (Castillo-Saavedra et al., 2016; Lefaucheur et al., 2017).

### Safety and tolerance

Our study did not find any serious complications of tDCS, and it was well-tolerated. Moreover, a recently published review observed no considerable adverse effects of tDCS (Brunoni et al., 2012). However, mild adverse effects of tDCS are comparatively pervasive, for instance, burning, tingling, itching near the electrodes, or paradoxical depression worsening. The current criteria employed in clinical investigations were demonstrated safe in one animal safety study. The reason was that brain lesions were just experimentally induced when mice were subjected to cathode stimulation, almost 100 times higher than those used in hospital trials (Liebetanz et al., 2009). However, clinical utilization of tDCS involves the repetitive and routine application of tDCS. These stimulants may induce side effects associated with successive stimulation. In addition, embedding saline (15–140 mm) solution and anesthetic ointment in the therapeutic sponge electrodes can prevent adverse effects (Brunoni et al., 2012). Therefore, later investigations are required for adverse effects during tDCS intervention.

## Strengths and limitations

Regarding study strengths, this study is the first meta-analysis on the effectiveness of tDCS on chronic pain, depressive and anxiety symptoms. We highlighted the psychological factors that deserve attention in cases suffering from chronic pain and the potential efficacy of tDCS in the treatment of comorbidities. Most included studies in our analysis presented low risk of bias. Our results support the associations among many physical and mental symptoms.

Several limitations of this meta-analysis were as follows: (1) The quantity of members in depression and anxiety was moderately little, so caution is required when deciphering the connection between chronic pain and disorder mood, as underpowered RCTs might diminish the possibility of distinguishing a genuine impact and the probability of statistically significant outcomes. (2) We included a sample of patients experiencing fluctuating levels of depression or anxiety in the current research given that we mainly scrutinized changes in pain intensity and keep a homogenous pain intensity at baseline. Psychiatric issues are regularly profoundly comorbid: anxiety and depression often coexist in chronic pain patients. Whether the efficacy of tDCS on chronic pain patients with mood disorders is affected by the degree of mood disorders would be a significant element to address in further tDCS investigations. (3) Similar to chronic pain, acute pain patients are also vulnerable to anxiety and depression comorbidities (Doan et al., 2015; Michaelides and Zis, 2019). Notably, tDCS has also been applied in the management of acute pain (Hamner et al., 2015; Hosseini Amiri et al., 2016). Thus, future studies should consider further exploring the efficacy of tDCS in patients with acute pain associated with anxiety or depression.

## Conclusion

The present meta-analytic review proposes that tDCS might be considered a short-term therapy for chronic pain patients experiencing depression or anxiety. It has a reasonable bearableness profile, which would be a powerful option for patients who don't profit from existing pharmacological and additionally mental medicines. Detailed tDCS parameters (e.g., 2-mA intervention over 20 min/session, treatment more than 10 sessions on M1) and clinical characteristics (neuropathic pain) may augment the function of tDCS. We did not recommend involving tDCS as a sole clinical therapy but we propose that it can be a consideration in the relief of short-lasting chronic pain accompanied by a psychological disorder. Persistent pain is considered an illness that cannot be cured but can be managed. Thus, the treatment of chronic pain is aimed at the illness, rather than at the disease. Further investigations of tDCS in comorbid patients with mood disorders and chronic pain are required, particularly those that evaluate large samples and address criteria that influence a methodology's effectivity on clinical information, particularly rates of remission and relapse. Existing evidence does not show that tDCS is effective in mid-to-late follow-up, but future evidence may change this conclusion, and the theoretical and mechanistic basis of tDCS as a pain comorbidity treatment is worthy of careful study.

## Data availability statement

The original contributions presented in the study are included in the article/Supplementary material, further inquiries can be directed to the corresponding authors.

## Author contributions

Y-RW and JS wrote the first draft of the manuscript and managed the systematic literature searches. Y-TL, Z-YH, and XJ performed statistical analysis using Stata. RW corrected the English style. Y-LW, X-QW, and Y-YL supervised the study and critically revised the manuscript for important intellectual content. All authors contributed to the final manuscript and all approved it.

## Funding

This work was supported by the grant from the National Key R&D Program of China (Grant No. 2020YFC2007700) and the Guangdong Hopson-Pearl River Education Development Foundation (Grant No. H20190116202012724).

## Conflict of interest

The authors declare that the research was conducted in the absence of any commercial or financial relationships that could be construed as a potential conflict of interest.

## Publisher's note

All claims expressed in this article are solely those of the authors and do not necessarily represent those of their affiliated organizations, or those of the publisher, the editors and the reviewers. Any product that may be evaluated in this article, or claim that may be made by its manufacturer, is not guaranteed or endorsed by the publisher.

## Supplementary material

Supplementary Figure S1 Forest plot showing SMDs in pain intensity scores from data of short-term and medium-term studies. **(A)** Sub-analysis of pain intensity scores by the number of tDCS stimulation sessions in short-term studies. **(B)** Sub-analysis of pain intensity scores by the number of tDCS stimulation sessions in medium-term studies. CI, confidence interval; SMD, standard mean difference; tDCS, transcranial direct current stimulation; M1, motor cortex; DLPFC, dorsolateral prefrontal cortex; a, motor cortex; b, dorsolateral prefrontal cortex, <5 Sessions <5 tDCS treatment sessions, 5–10 sessions 5–10 tDCS treatment sessions, >10 sessions more than 10 tDCS treatment sessions.

Supplementary Figure S2 Forest plot showing overall effect sizes (Hedges' g) of real tDCS on pain intensity within studies from long-term data. These plots show the pooled SMD (large diamond shape) and *I*^2^ resulting from the meta-analysis. CI, confidence interval; SMD, standard mean difference; tDCS, transcranial direct current stimulation.

Supplementary Figure S3 Forest plot showing overall effect sizes (Hedges' g) of real tDCS on depression scores within studies from long-term data. These plots show the pooled SMD (large diamond shape) and *I*^2^ resulting from the meta-analysis. CI, confidence interval; SMD, standard mean difference; tDCS, transcranial direct current stimulation; a, motor cortex; b, dorsolateral prefrontal cortex; c, operculo-insular cortex.

Supplementary Figure S4 Forest plot showing overall effect sizes (Hedges' g) of real tDCS on anxiety scores within studies from long-term data. These plots show the pooled SMD (large diamond shape) and *I*^2^ resulting from the meta-analysis. CI, confidence interval; SMD, standard mean difference; tDCS, transcranial direct current stimulation; a, motor cortex; b, dorsolateral prefrontal cortex; c, operculo-insular cortex.

Supplementary Figure S5 Funnel plots with standard errors plotted against effect sizes for determining publication bias in chronic pain studies limited stimulus to the motor cortex at short-term follow-up.

Supplementary Figure S6 Funnel plots with standard errors plotted against effect sizes for determining publication bias in chronic pain intensity at medium-term follow-up.

Supplementary Figure S7 Funnel plots with standard errors plotted against effect sizes for determining publication bias in chronic pain intensity at short-term follow-up.

Supplementary material 1 Search strategy.

Supplementary material 2 Methods.

Supplementary material 3 Results.

Supplementary material 4 Active tDCS compared to Sham tDCS for treating chronic pain with depression or anxiety.

Supplementary material 5 Abbreviations.
