# Supplementary figures and images for: Is transcranial direct current stimulation beneficial for treating pain, depression, and anxiety symptoms in patients with chronic pain? A systematic review and meta-analysis
Source: Front Mol Neurosci. 2022 Dec 1;15:1056966. doi: 10.3389/fnmol.2022.1056966 (PMC9752114; doi:10.3389/fnmol.2022.1056966)

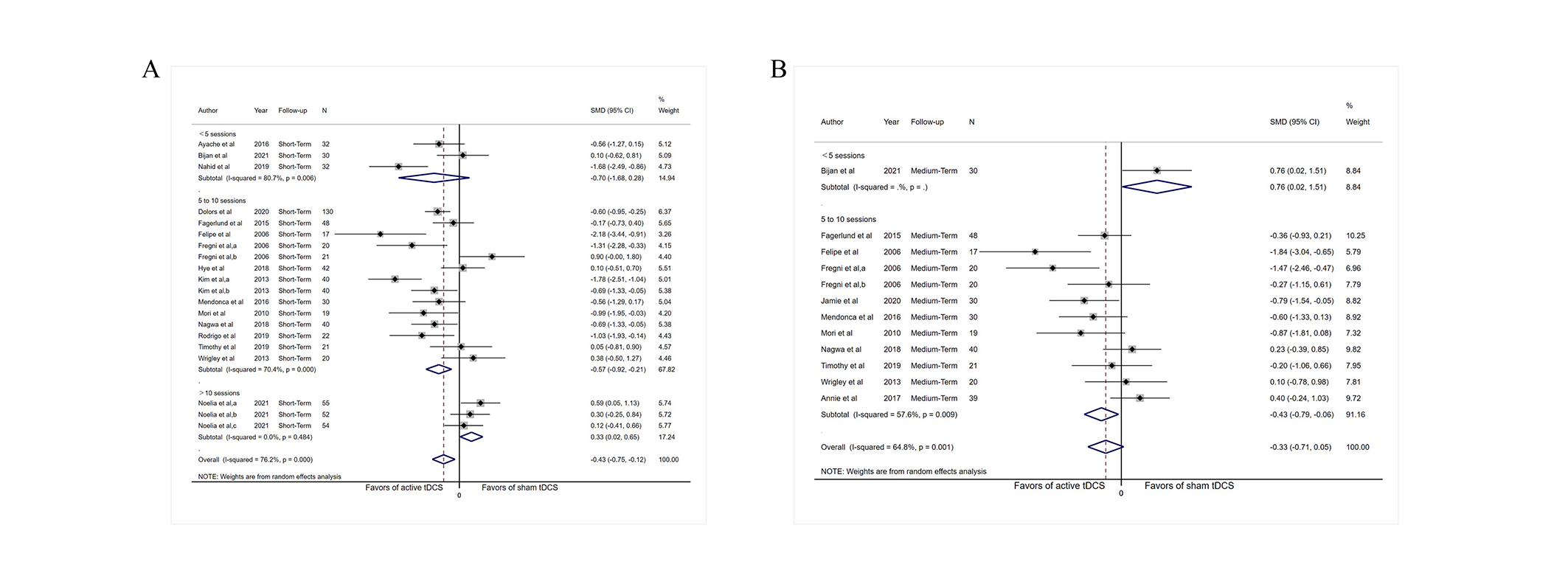

Supplement: Supplementary Figure S1 — Forest plot showing SMDs in pain intensity scores from data of short-term and medium-term studies. (A) Sub-analysis of pain intensity scores by the number of tDCS stimulation sessions in short-term studies. (B) Sub-analysis of pain intensity scores by the number of tDCS stimulation sessions in medium-term studies. CI, confidence interval; SMD, standard mean difference; tDCS, transcranial direct current stimulation; M1, motor cortex; DLPFC, dorsolateral prefrontal cortex; a, motor cortex; b, dorsolateral prefrontal cortex, <5 Sessions <5 tDCS treatment sessions, 5–10 sessions 5–10 tDCS treatment sessions, >10 sessions more than 10 tDCS treatment sessions. [file Image_1.TIF]

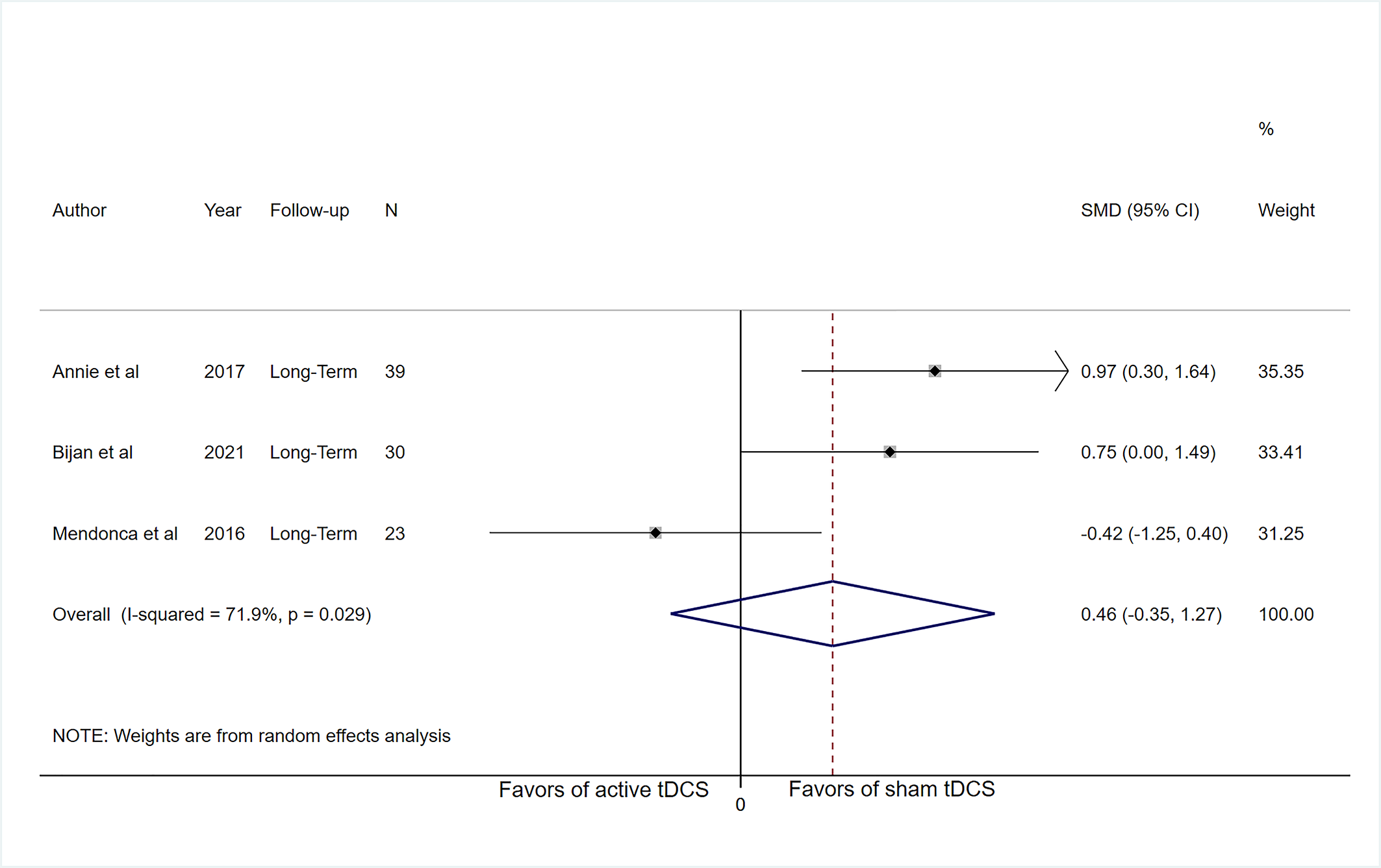

Supplement: Supplementary Figure S2 — Forest plot showing overall effect sizes (Hedges' g) of real tDCS on pain intensity within studies from long-term data. These plots show the pooled SMD (large diamond shape) and I2 resulting from the meta-analysis. CI, confidence interval; SMD, standard mean difference; tDCS, transcranial direct current stimulation. [file Image_2.TIF]

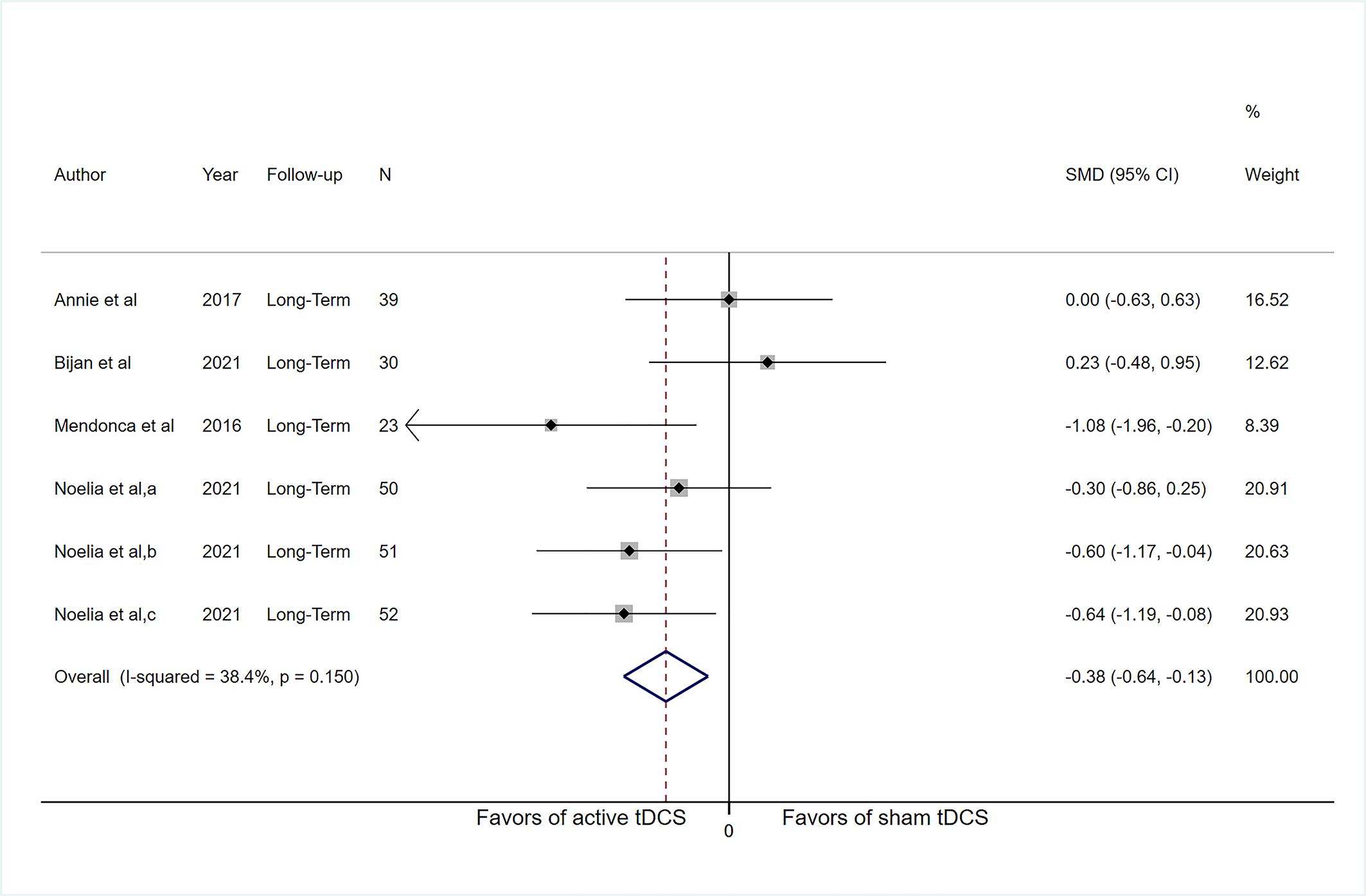

Supplement: Supplementary Figure S3 — Forest plot showing overall effect sizes (Hedges' g) of real tDCS on depression scores within studies from long-term data. These plots show the pooled SMD (large diamond shape) and I2 resulting from the meta-analysis. CI, confidence interval; SMD, standard mean difference; tDCS, transcranial direct current stimulation; a, motor cortex; b, dorsolateral prefrontal cortex; c, operculo-insular cortex. [file Image_3.TIF]

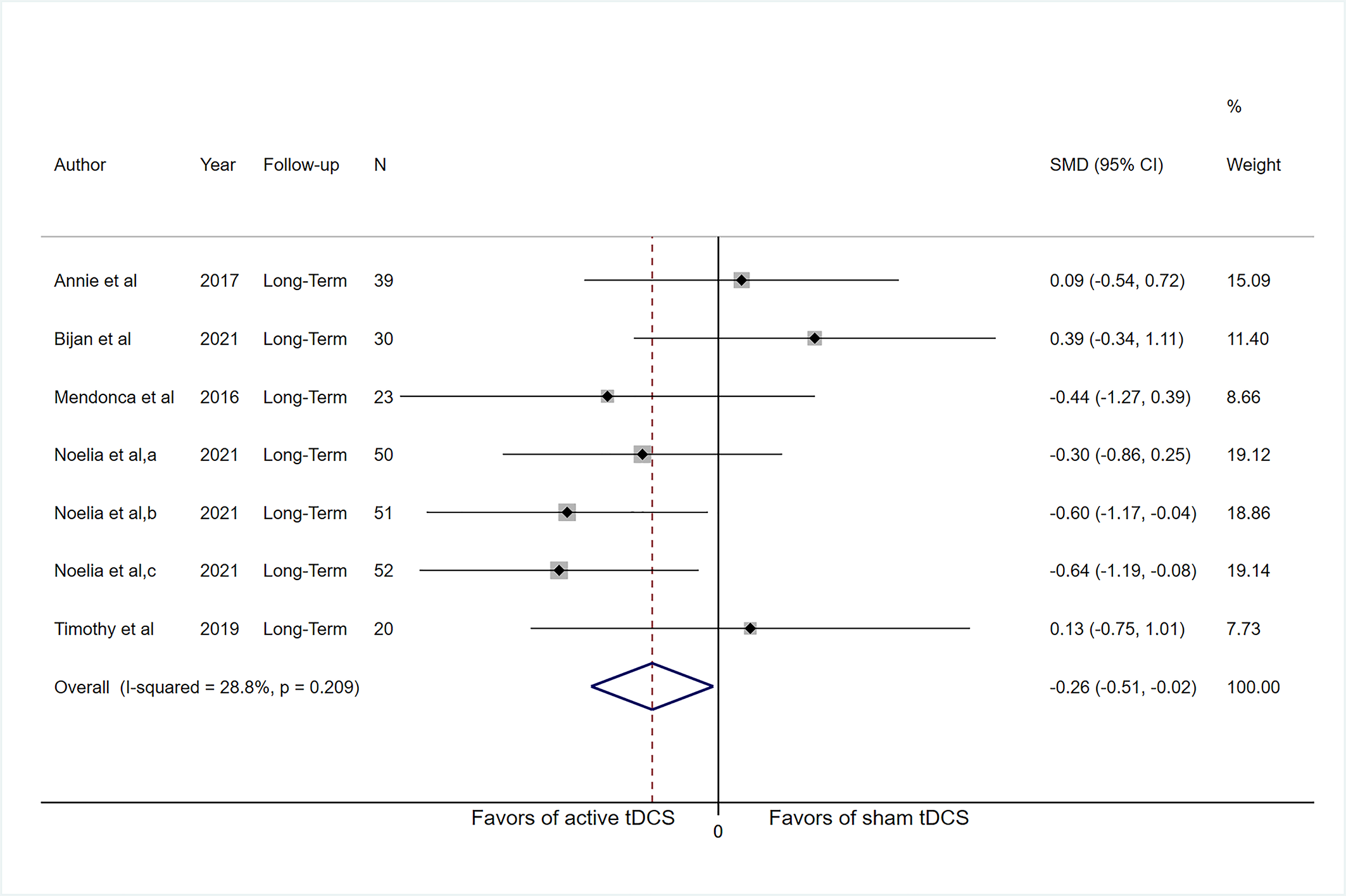

Supplement: Supplementary Figure S4 — Forest plot showing overall effect sizes (Hedges' g) of real tDCS on anxiety scores within studies from long-term data. These plots show the pooled SMD (large diamond shape) and I2 resulting from the meta-analysis. CI, confidence interval; SMD, standard mean difference; tDCS, transcranial direct current stimulation; a, motor cortex; b, dorsolateral prefrontal cortex; c, operculo-insular cortex. [file Image_4.TIF]

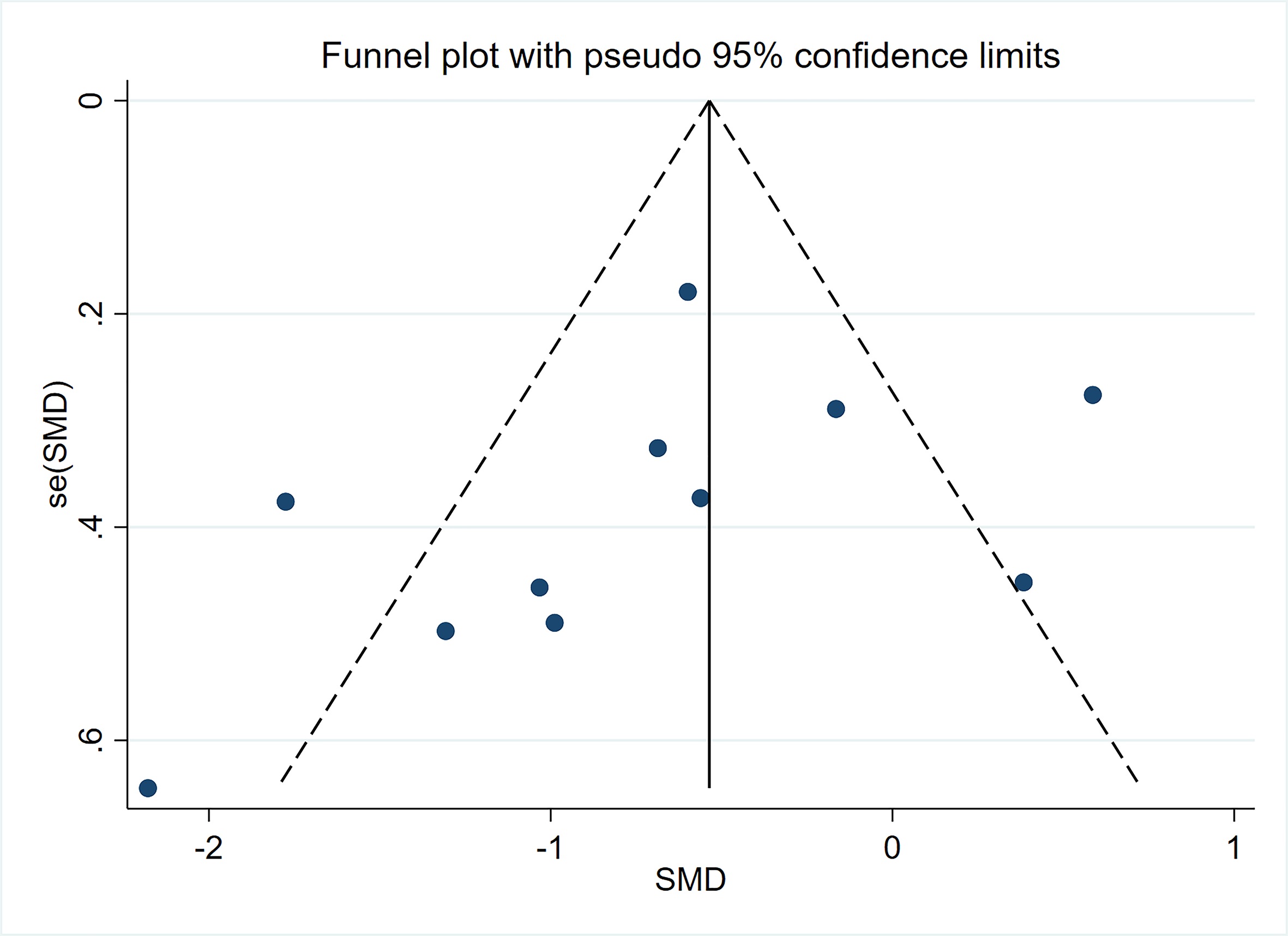

Supplement: Supplementary Figure S5 — Funnel plots with standard errors plotted against effect sizes for determining publication bias in chronic pain studies limited stimulus to the motor cortex at short-term follow-up. [file Image_5.TIF]

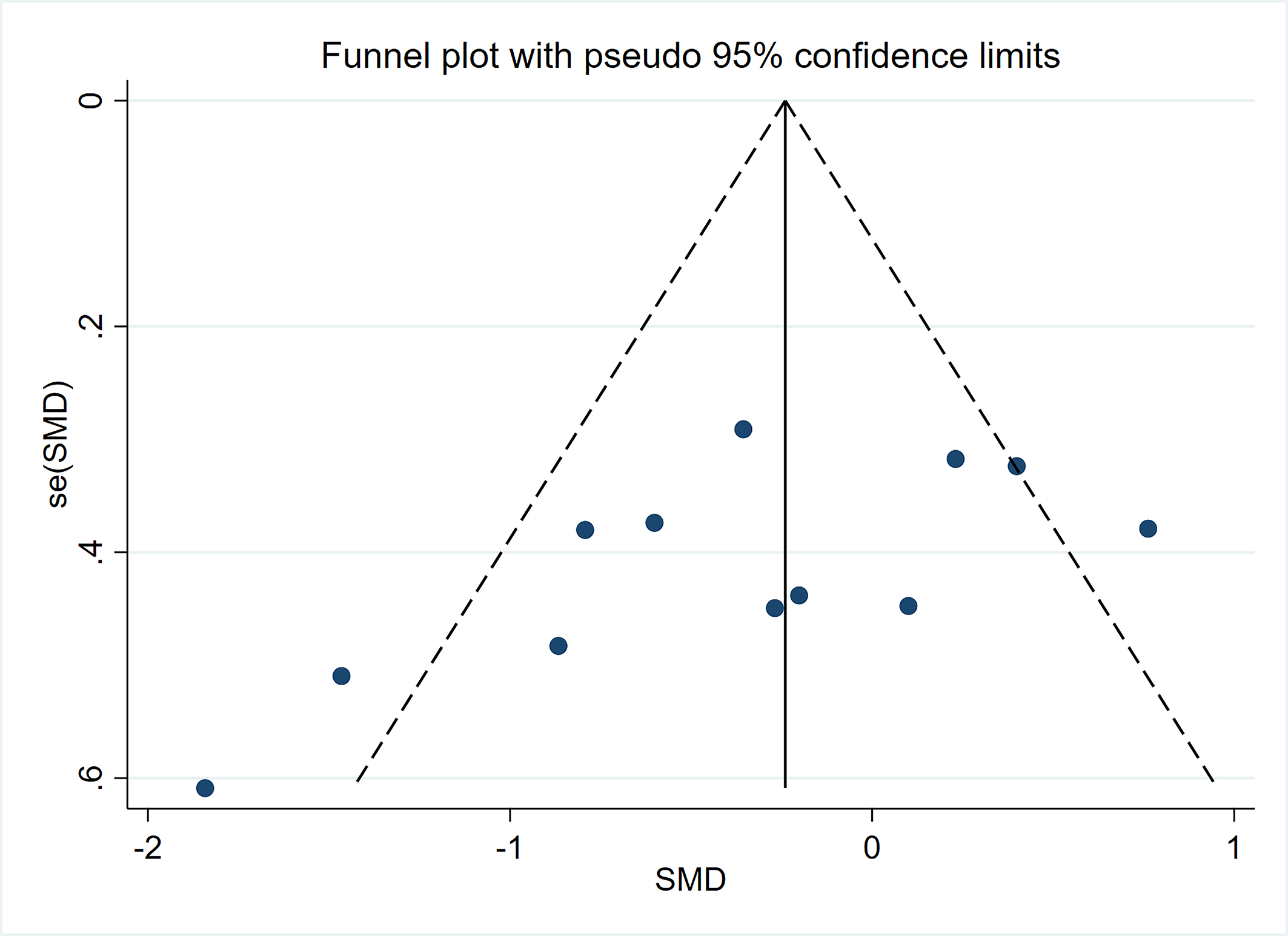

Supplement: Supplementary Figure S6 — Funnel plots with standard errors plotted against effect sizes for determining publication bias in chronic pain intensity at medium-term follow-up. [file Image_6.TIF]

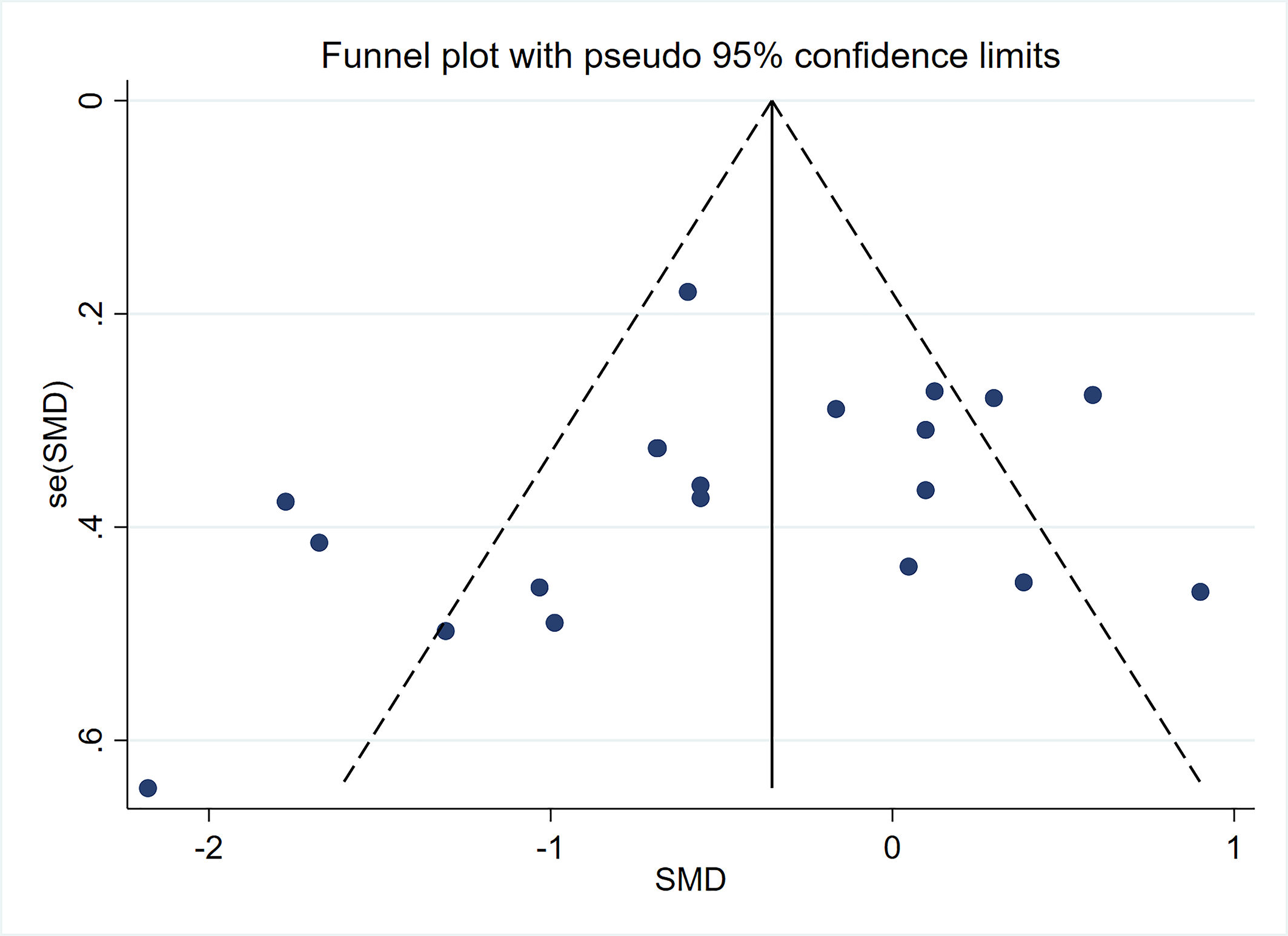

Supplement: Supplementary Figure S7 — Funnel plots with standard errors plotted against effect sizes for determining publication bias in chronic pain intensity at short-term follow-up. [file Image_7.TIF]
