# Supplementary material for: Is transcranial direct current stimulation beneficial for treating pain, depression, and anxiety symptoms in patients with chronic pain? A systematic review and meta-analysis
Source: Front Mol Neurosci. 2022 Dec 1;15:1056966. doi: 10.3389/fnmol.2022.1056966 (PMC9752114; doi:10.3389/fnmol.2022.1056966)
Supplement: Supplementary material 1 — Search strategy. [file Data_Sheet_1.docx]

**Supplementary material 1-Search strategy**

**Cochrane library**

#1: MeSH descriptor: [Pain] explode all trees

#2: (chronic* or back or musculoskel* or intractabl* or neuropath* or phantom limb or fantom limb or neck or myofasc* or "temporomandib* joint" or "temperomandib* joint" or "tempromandib* joint" or central or (post next stroke) or complex or regional or "spinal cord") near/4 pain*:ti,ab,kw  (Word variations have been searched)

#3: (sciatica or back‐ache or back*ache or lumbago or fibromyalg* or (trigemin* near/2 neuralg*) or (herp* near/2 neuralg*) or (diabet* near/2 neuropath*) or (reflex near/4 dystroph*) or (sudeck* near/2 atroph*) or causalg* or whip‐lash or whip*lash or polymyalg* or (failed back near/4 surg*) or (failed back near/4 syndrome*)):ti,ab,kw  (Word variations have been searched)

#4: #1 OR #2 OR #3

#5: MeSH descriptor: [Electric Stimulation Therapy] explode all trees

#6: MeSH descriptor: [Electric Stimulation] explode all trees

#7: MeSH descriptor Electrodes explode all trees

#8: (transcranial NEAR/5 “direct current” NEAR/5 stimulation):ti,ab,kw (Word variations have been searched)

#9: (transcranial NEAR/5 DC NEAR/5 stimulation):ti,ab,kw (Word variations have been searched)

#10: (transcranial NEAR/5 electric* NEAR/5 stimulation):ti,ab,kw (Word variations have been searched)

#11: (tDCS or A‐tDCS or C‐tDCS or S‐tDCS or electrode* or anode or anodes or anodal or cathode or cathodes or cathodal):ti,ab,kw (Word variations have been searched)

#12: #5 OR #6 OR #7 OR #8 OR #9 OR #10 OR #11

#13: MeSH descriptor: [Depression] explode all trees

#14: MeSH descriptor: [Depressive Disorder] explode all trees

#15: MeSH descriptor: [Anxiety] explode all trees

#16: MeSH descriptor: [Emotions] explode all trees

#17: ((Depression) OR (Depressiveness) OR (Depressive Symptoms) OR (Depressive Symptom) OR (Emotional Depression) OR (Emotional Depressions) OR (Depressive Disorders) OR (Depressive Neuroses) OR (Depressive Neurosis) OR (Endogenous Depression) OR (Endogenous Depressions) OR (Depressive Syndrome) OR (Depressive Syndromes) OR (Neurotic Depression) OR (Neurotic Depressions) OR (Melancholia) OR (Melancholias) OR (Unipolar Depression) OR (Unipolar Depressions) OR (dysthymia)):ti,ab,kw

#18: ((Angst) OR (Nervousness) OR (Hypervigilance) OR (Anxiousness) OR (Social Anxiety) OR (Social Anxieties)):ti,ab,kw

#19: #13 OR #14 OR #15 OR #16 OR #17 OR #18

#20: #4 AND #12 AND #19

**PubMed**

#1: ("Chronic Pain"[Mesh]) OR (Chronic Pains) OR (Widespread Chronic Pain)

#2: (sciatica OR back‐ache OR back* ache OR lumbago OR fibromyalg* OR "reflex dystroph*" OR "sudeck* atroph*" OR causalg* OR whip‐lash OR whip* lash OR polymyalg* OR "failed back surg*" OR "failed back syndrome*") OR ((tempromandib* joint*) AND (pain*)) OR ((temperomandib* joint*) AND (pain*)) OR ((temporomandib* joint*) AND (pain*)) OR ((chronic* OR back OR musculoskel* OR intractabl* OR neuropath* OR phantom limb OR fantom limb OR neck OR myofasc* OR central OR post* stroke OR complex OR regional OR spinal cord) AND pain*))

#3: #1OR#2

#4: (Depressions) OR (Depressiveness) OR (Depressive Symptoms) OR (Depressive Symptom) OR (Emotional Depression) OR (Depressive Disorders) OR (Depressive Neuroses) OR (Depressive Neurosis) OR (Endogenous Depression) OR (Endogenous Depressions) OR (Depressive Syndrome) OR (Depressive Syndromes) OR (Neurotic Depression) OR (Neurotic Depressions) OR (Melancholia) OR (Melancholias) OR (Unipolar Depression) OR (Unipolar Depressions) OR (dysthymia) OR ("Depression"[Mesh]) OR ("Depressive Disorder"[Mesh])

#5: (Angst) OR (Nervousness) OR (Hypervigilance) OR (Anxiousness) OR (Social Anxiety) OR (Social Anxieties) OR ("Anxiety"[Mesh]) OR ("Depressive Disorder"[Mesh]) OR ("Emotions"[Mesh])

#6: #4OR#5

#7: (tDCS) OR (Cathodal Stimulation Transcranial Direct Current Stimulation) OR (Cathodal Stimulation tDCS) OR (Cathodal Stimulation tDCSs) OR (Transcranial Random Noise Stimulation) OR (Transcranial Alternating Current Stimulation) OR (Transcranial Electrical Stimulation) OR (Transcranial Electrical Stimulations) OR (Anodal Stimulation Transcranial Direct Current Stimulation) OR (Anodal Stimulation tDCS) OR (Anodal Stimulation tDCSs) OR (Repetitive Transcranial Electrical Stimulation) OR ("Transcranial Direct Current Stimulation"[Mesh])

#8: #3AND#6AND#7

**WOS**

#1: (tDCS) OR (Cathodal Stimulation Transcranial Direct Current Stimulation) OR (Cathodal Stimulation tDCS) OR (Transcranial Random Noise Stimulation) OR (Transcranial Alternating Current Stimulation) OR (Transcranial Electrical Stimulation) OR (Transcranial Electrical Stimulations) OR (Anodal Stimulation Transcranial Direct Current Stimulation) OR (Anodal Stimulation tDCS) OR (Repetitive Transcranial Electrical Stimulation) OR (Transcranial Direct Current Stimulation)

#2: (Depressions) OR (Depressive Symptoms) OR (Depressive Symptom) OR (Emotional Depression) OR (Emotional Depressions) OR (Depressiveness) OR (Depressive Disorders) OR (Depressive Neuroses) OR (Depressive Neurosis) OR (Endogenous Depression) OR (Endogenous Depressions) OR (Depressive Syndrome) OR (Depressive Syndromes) OR (Neurotic Depression) OR (Neurotic Depressions) OR (Melancholia) OR (Melancholias) OR (Unipolar Depression) OR (Unipolar Depressions) OR (Depression) OR (Depressive Disorder) OR (dysthymia)

#3: (Angst) OR (Nervousness) OR (Hypervigilance) OR (Anxiousness) OR (Social Anxiety) OR (Social Anxieties) OR (Anxiety) OR (Emotions)

#4: ((Chronic Pain) OR (Chronic Pains) OR (Widespread Chronic Pain) OR (Widespread Chronic Pains) OR (sciatica OR back‐ache OR back* ache OR lumbago OR fibromyalg* OR "reflex dystroph*" OR "sudeck* atroph*" OR causalg* OR whip‐lash OR whip* lash OR polymyalg* OR "failed back surg*" OR "failed back syndrome*") OR ((tempromandib* joint*) AND (pain*) ) OR ((temperomandib* joint*) AND (pain*) )) OR ((temporomandib* joint*) AND (pain*))OR ((chronic* OR back OR musculoskel* OR intractabl* OR neuropath* OR phantom limb OR fantom limb OR neck OR myofasc* OR central OR post* stroke OR complex OR regional OR spinal cord) AND (pain*))

#5: #3 OR #2

#6: #5 AND #4

#7: #6 AND #1

**EMBASE**

#1: 'pain'/exp

#2: 'chronic pains':ti,ab,kw OR 'widespread chronic pain':ti,ab,kw OR 'widespread chronic pains':ti,ab,kw OR sciatica:ti,ab,kw OR back‐ache:ti,ab,kw OR 'back* ache':ti,ab,kw OR lumbago:ti,ab,kw OR fibromyalg*:ti,ab,kw OR 'reflex dystroph*':ti,ab,kw OR 'sudeck* atroph*':ti,ab,kw OR causalg*:ti,ab,kw OR whip‐lash:ti,ab,kw OR 'whip* lash':ti,ab,kw OR polymyalg*:ti,ab,kw OR 'failed back surg*':ti,ab,kw OR 'failed back syndrome*':ti,ab,kw OR ('tempromandib* joint*':ti,ab,kw AND pain*:ti,ab,kw) OR ('temperomandib* joint*':ti,ab,kw AND pain*:ti,ab,kw) OR ('temporomandib* joint*':ti,ab,kw AND pain*:ti,ab,kw) OR ((chronic*:ti,ab,kw OR back:ti,ab,kw OR musculoskel*:ti,ab,kw OR intractabl*:ti,ab,kw OR neuropath*:ti,ab,kw OR 'phantom limb':ti,ab,kw OR 'fantom limb':ti,ab,kw OR neck:ti,ab,kw OR myofasc*:ti,ab,kw OR central:ti,ab,kw OR 'post* stroke':ti,ab,kw OR complex:ti,ab,kw OR regional:ti,ab,kw OR 'spinal cord':ti,ab,kw) AND pain*:ti,ab,kw)

#3: #1OR#2

#4:depressions:ti,ab,kw OR 'depressive symptoms':ti,ab,kw OR 'depressive symptom':ti,ab,kw OR 'emotional depression':ti,ab,kw OR 'emotional depressions':ti,ab,kw OR 'depressive disorders':ti,ab,kw OR 'depressive neuroses':ti,ab,kw OR 'depressive neurosis':ti,ab,kw OR 'endogenous depression':ti,ab,kw OR 'endogenous depressions':ti,ab,kw OR 'depressive syndrome':ti,ab,kw OR 'depressive syndromes':ti,ab,kw OR 'neurotic depression':ti,ab,kw OR 'neurotic depressions':ti,ab,kw OR melancholia:ti,ab,kw OR melancholias:ti,ab,kw OR 'unipolar depression':ti,ab,kw OR 'unipolar depressions':ti,ab,kw OR depression:ti,ab,kw OR 'depressive disorder':ti,ab,kw OR 'depressiveness':ti,ab,kw OR dysthymia:ti,ab,kw

#5: 'depression'/exp

#6: 'anxiety disorder'/exp

#7: 'anxiety'/exp

#8: 'emotion'/exp

#9: angst:ti,ab,kw OR nervousness:ti,ab,kw OR hypervigilance:ti,ab,kw OR anxiousness:ti,ab,kw OR 'social anxiety':ti,ab,kw OR 'social anxieties':ti,ab,kw OR anxiety:ti,ab,kw

#10: #4 OR#5OR#6OR#7OR#8OR#9

#11: 'transcranial direct current stimulation'/exp

#12:tdcs:ti,ab,kw OR 'cathodal stimulation transcranial direct current stimulation':ti,ab,kw OR 'cathodal stimulation tdcs':ti,ab,kw OR 'transcranial random noise stimulation':ti,ab,kw OR 'transcranial alternating current stimulation':ti,ab,kw OR 'transcranial electrical stimulation':ti,ab,kw OR 'transcranial electrical stimulations':ti,ab,kw OR 'anodal stimulation transcranial direct current stimulation':ti,ab,kw OR 'anodal stimulation tdcs':ti,ab,kw OR 'repetitive transcranial electrical stimulation':ti,ab,kw OR 'transcranial direct current stimulation':ti,ab,kw

#13: #11OR#12

#14: #3 AND #10 AND #13
