# Supplementary material for: Is transcranial direct current stimulation beneficial for treating pain, depression, and anxiety symptoms in patients with chronic pain? A systematic review and meta-analysis
Source: Front Mol Neurosci. 2022 Dec 1;15:1056966. doi: 10.3389/fnmol.2022.1056966 (PMC9752114; doi:10.3389/fnmol.2022.1056966)
Supplement: Supplementary material 2 — Methods. [file Data_Sheet_2.docx]

**Supplementary material 2-Methods**

**The standard information extraction form contained the following items:**

- Subject characteristics: age; gender; duration of pain; pain conditions; sample size; country of origin; baseline pain intensity; and baseline depression or anxiety
- Study design: parallel or cross design; control paradigm; randomization; blinding; other interventions.
- tDCS parameters: stimulation site, mode, frequency, intensity, duration
- Outcome measurements: pain intensity, the symptoms of depression and anxiety (mean ± SD) on the baseline and post-stimulation for the calculation of the mean difference between pre-and post-stimulation scores for the active tDCS and control groups; secondary outcomes included safety. The calculational methods of the mean and SD of the difference values are included in supplementary materials.

When the outcome was expressed only as a graph, Engauge Digitizer 10.8 (http://markummitchell.github.io/engauge-digitizer/) was used to extract the required data. When the raw data were not sufficiently extracted, we contacted the authors of the studies; the RevMan calculator was used to convert them to means and SDs when the standard errors (SEs), confidence intervals (CIs), or IQRs were supplied rather than means and SDs.

**Data extraction before and after treatment.**

|  | Pre- | Post- | Difference |
| --- | --- | --- | --- |
| tDCS group (Sample size,$N_{r}$) | $M_{r,pre-}$ | $M_{r,post-}$ | $\text{M}_{\text{r,difference}}\text{=}\text{M}_{\text{r,post-}}\text{-}\text{M}_{\text{r,pre-}}$ |
|  | ${SD}_{r,pre-}$ | ${SD}_{r,post-}$ | ${SD}_{r,difference}=\sqrt{{SD}_{r,pre-}^{2}+{SD}_{r,post-}^{2} \left( 2 \times0 . 5 \times{SD}_{r,pre-}\times{SD}_{r,post-} \right)}$ |
| Control group (Sample size,$N_{c}$) | $M_{c,pre-}$ | $M_{c,post-}$ | $M_{c,difference}=M_{c,post-}-M_{c,pre-}$ |
|  | ${SD}_{c,pre-}$ | ${SD}_{c,post-}$ | ${SD}_{c,difference}=\sqrt{{SD}_{c,pre-}^{2}+{SD}_{c,post-}^{2} \left( 2 \times0 . 5 \times{SD}_{c,pre-}\times{SD}_{c,post-} \right)}$ |
