# Supplementary material for: Is transcranial direct current stimulation beneficial for treating pain, depression, and anxiety symptoms in patients with chronic pain? A systematic review and meta-analysis
Source: Front Mol Neurosci. 2022 Dec 1;15:1056966. doi: 10.3389/fnmol.2022.1056966 (PMC9752114; doi:10.3389/fnmol.2022.1056966)
Supplement: Supplementary material 3 — Results. [file Data_Sheet_3.docx]

**Supplementary material 3-Results**

**Results**

***Overview***

Nine studies recruited patients with non-neuropathic pain, six with fibromyalgia (Fregni et al., 2006b; Fagerlund et al., 2015; Mendonca et al., 2016; Yoo et al., 2018; Forogh et al., 2021; Samartin-Veiga et al., 2022) and three with chronic low back pain (Mariano et al., 2019; Shiasy et al., 2020; McPhee and Graven-Nielsen, 2021). Eight studies enrolled participants with neuropathic pain, seven studies (Fregni et al., 2006a; Mori et al., 2010; Wrigley et al., 2013; Ayache et al., 2016; Young et al., 2020; Gunduz et al., 2021; Soler et al., 2021) enrolled those with central neuropathic pain and one study (Kim et al., 2013) enrolled those with peripheral neuropathic in diabetes. Six studies included individuals with visceral pain, one included those with provoked vestibulodynia (Morin et al., 2017), one included those with visceral pain and hepatocellular carcinoma (Ibrahim et al., 2018), one included those with chronic pelvic pain (Divandari et al., 2019) and two included those with chronic abdominal pain (Dutra et al., 2020; Pegado et al., 2020).

Most studies evaluated pain intensity at the short-term (< 1week post-intervention) follow-up stage except (Morin et al., 2017; Young et al., 2020). Eleven studies presented medium-term results achievements (1–6 weeks post-intervention). Only three studies (Mendonca et al., 2016; Morin et al., 2017; Forogh et al., 2021) gathered information at long-term (> 6 weeks post-intervention) follow-up. Ten studies measured depression scores at the short-term stage after the end of interventions. Nine studies used measures of depression at the medium-term stage. Six studies used measures of depression at the long-term stage. Six studies (Mori et al., 2010; Yoo et al., 2018; Mariano et al., 2019; Shiasy et al., 2020; Young et al., 2020; Forogh et al., 2021) did not report data with regards to adverse events.

Four investigations applied stimulation on the DLPFC in one experimental group (Fregni et al., 2006b; Ayache et al., 2016; Yoo et al., 2018; Forogh et al., 2021). Thirteen studies of tDCS stimulated the M1. Two studies (Fregni et al., 2006b; Kim et al., 2013) stimulated M1 and DLPFC in experimental groups. One study (Mariano et al., 2019) stimulated dorsal anterior cingulate cortex (dACC). One study (McPhee and Graven-Nielsen, 2021) stimulated mPFC. One study (Divandari et al., 2019) stimulated M1 and DLPFC simultaneously. One study (Yoo et al., 2018) stimulated bilateral DLPFC, simultaneously. Four studies specifically investigated the utilization of tDCS connected with other interventions, including aerobic exercise (Mendonca et al., 2016), attention bias modification (Shiasy et al., 2020), visual illusion (Soler et al., 2021) and mirror therapy (Gunduz et al., 2021). One single study (Mariano et al., 2019) applied multiple sessions of cathodal tDCS stimulation over the left dACC.

Twenty studies provided a current intensity of 2 mA once a day for 20 minutes. Only one study delivered 2 mA once a day for 30 minutes (Ibrahim et al., 2018) and one study delivered 0.3 mA once a day for 20 minutes (Divandari et al., 2019). Among all studies, the number and frequency of stimulus sessions varied significantly, from one session to up to 15 sessions.

Data on the severity of chronic pain, depression, and anxiety were obtained from 18 studies. Four studies (Dutra et al., 2020; Shiasy et al., 2020; Gunduz et al., 2021; McPhee and Graven-Nielsen, 2021) were included in the qualitative analysis, and the details can be found in Table1. We were incapable of including data from (Dutra et al., 2020; Shiasy et al., 2020; Gunduz et al., 2021; McPhee and Graven-Nielsen, 2021) because these data were not reported.

***Risk of bias***

The risk of bias was different across all study criteria. The risk of bias in random sequence generation was generally unclear because the four RCTs (Yoo et al., 2018; Divandari et al., 2019; Samartin-Veiga et al., 2022; Soler et al., 2021) do not specify or describe the randomization method used. The remaining 18 studies were considered to be at low risk of bias in this domain. We judged 11 studies that did not explicitly describe concealment of allocation.

All studies attempted to blind participants. We judged one study (Shiasy et al., 2020) at unclear risk of bias as the method of blinding was not described. As there is no explanation of how to cover up, we, therefore, judged one study at unclear risk of bias. One study according to unsuccessful blinding and was rated at high risk (Divandari et al., 2019). Different four studies were rated at high risk because of the control group setting. (Yoo et al., 2018; Young et al., 2020; Forogh et al., 2021; Soler et al., 2021). We have a tendency to judge 17 studies at low risk of bias, three studies (Mariano et al., 2019; Shiasy et al., 2020; Gunduz et al., 2021) to be at unclear risk of bias for blinding of assessors, and a pair of studies (Yoo et al., 2018; Young et al., 2020) at high risk of bias, as they clearly claimed that assessors weren't unsighted. The rest of the studies were rated at low risk of bias.

We assessed four studies (Kim et al., 2013; Mendonca et al., 2016; Morin et al., 2017; Samartin-Veiga et al., 2022) as having a high risk of bias for incomplete outcome data. In the study (Morin et al., 2017), one participant in the active group was not considered for analysis due to the absence of a penetration attempt. (Kim et al., 2013) reported a 15% dropout rate and those participants seem to own been excluded from the analysis. (Mendonca et al., 2016) reported 13 participants (28%) withdrew from the study and excluded those participants from the analysis. Within the study by (Samartin-Veiga et al., 2022), 23.07% of participants discontinued the trial. We tend to assess two studies (Kim et al., 2013; Shiasy et al., 2020) as having a high risk of bias for selective coverage and one study as being at unclear risk of bias. We tend to assess the remaining 19 studies as having a low risk of bias for this criterion. We judged one study (Divandari et al., 2019) at unclear risk for other bias as two patients perceived that one session of their treatment was sham, whereas others could not distinguish between the sessions. The risk of bias in crossover studies indicated flaws related to the absence of sample size calculations, blinding of the participant, and assessor and allocation concealment.

***Publication bias***

We performed publication bias analysis for subgroups that containing 10 or more studies. The funnel plot was drawn to show the distribution of the studies. The distribution was relatively symmetrical, demonstrating no robust evidence of publication bias, except in the short-term depression effects of tDCS (Supplementary Figure S7). The publication bias assessment of articles from other groups is shown in Supplementary Figure S5 and Figure S6. Egger’s test corroborated this finding (P = 0.048). We further corrected the results through trim and fill analysis, which estimated four missing studies. The sum of the estimates and 95% CI was −0.396 (−0.547, −0.245) and −0.382 (−0.558, −0.206), respectively, after trimming and filling. No change in the results before and after the trim and fill analysis was observed, showing that they were robust.

**Reference**

Ayache, S.S., Palm, U., Chalah, M.A., Al-Ani, T., Brignol, A., Abdellaoui, M., et al., 2016. Prefrontal tDCS decreases pain in patients with multiple sclerosis. Front. Neurosci. 10**,** 147. https://doi.org/10.3389/fnins.2016.00147.

Divandari, N., Manshadi, F.D., Shokouhi, N., Vakili, M., and Jaberzadeh, S., 2019. Effect of one session of tDCS on the severity of pain in women with chronic pelvic pain. J. Bodyw Mov. Ther. 23**,** 678-682. https://doi.org/10.1016/j.jbmt.2017.12.014.

Dutra, L., Pegado, R., Silva, L.K., da Silva Dantas, H., Câmara, H.A., Silva-Filho, E.M., et al., 2020. Modulating anxiety and functional capacity with anodal tdcs over the left dorsolateral prefrontal cortex in primary dysmenorrhea. Int. J. Womens Health 12**,** 243-251. https://doi.org/10.2147/ijwh.S226501.

Fagerlund, A.J., Hansen, O.A., and Aslaksen, P.M., 2015. Transcranial direct current stimulation as a treatment for patients with fibromyalgia: a randomized controlled trial. Pain 156**,** 62-71. https://doi.org/10.1016/j.pain.0000000000000006.

Forogh, B., Haqiqatshenas, H., Ahadi, T., Ebadi, S., Alishahi, V., and Sajadi, S., 2021. Repetitive transcranial magnetic stimulation (rTMS) versus transcranial direct current stimulation (tDCS) in the management of patients with fibromyalgia: A randomized controlled trial. Neurophysiol Clin. 51**,** 339-347. https://doi.org/10.1016/j.neucli.2021.03.002.

Fregni, F., Boggio, P.S., Lima, M.C., Ferreira, M.J., Wagner, T., Rigonatti, S.P., et al., 2006a. A sham-controlled, phase II trial of transcranial direct current stimulation for the treatment of central pain in traumatic spinal cord injury. Pain 122**,** 197-209. https://doi.org/10.1016/j.pain.2006.02.023.

Fregni, F., Gimenes, R., Valle, A.C., Ferreira, M.J., Rocha, R.R., Natalle, L., et al., 2006b. A randomized, sham-controlled, proof of principle study of transcranial direct current stimulation for the treatment of pain in fibromyalgia. Arthritis Rheum. 54**,** 3988-3998. https://doi.org/10.1002/art.22195.

Gunduz, M.E., Pacheco-Barrios, K., Bonin Pinto, C., Duarte, D., Vélez, F.G.S., Gianlorenco, A.C.L., et al., 2021. Effects of combined and alone transcranial motor cortex stimulation and mirror therapy in phantom limb pain: A randomized factorial trial. Neurorehabil Neural. Repair. 35**,** 704-716. https://doi.org/10.1177/15459683211017509.

Ibrahim, N.M., Abdelhameed, K.M., Kamal, S.M.M., Khedr, E.M.H., and Kotb, H.I.M., 2018. Effect of transcranial direct current stimulation of the motor cortex on visceral pain in patients with hepatocellular carcinoma. Pain Med. 19**,** 550-560. https://doi.org/10.1093/pm/pnx087.

Kim, Y.J., Ku, J., Kim, H.J., Im, D.J., Lee, H.S., Han, K.A., et al., 2013. Randomized, sham controlled trial of transcranial direct current stimulation for painful diabetic polyneuropathy. Ann. Rehabil. Med. 37**,** 766-776. https://doi.org/10.5535/arm.2013.37.6.766.

Mariano, T.Y., Burgess, F.W., Bowker, M., Kirschner, J., Van't Wout-Frank, M., Jones, R.N., et al., 2019. Transcranial direct current stimulation for affective symptoms and functioning in chronic low back pain: A pilot double-blinded, randomized, placebo-controlled trial. Pain Med*.* 20**,** 1166-1177. https://doi.org/10.1093/pm/pny188.

McPhee, M.E., and Graven-Nielsen, T., 2021. Medial prefrontal high-definition transcranial direct current stimulation to improve pain modulation in chronic low back pain: A pilot randomized double-blinded placebo-controlled crossover trial. J. Pain 22**,** 952-967. https://doi.org/10.1016/j.jpain.2021.02.012.

Mendonca, M.E., Simis, M., Grecco, L.C., Battistella, L.R., Baptista, A.F., and Fregni, F., 2016. Transcranial direct current stimulation combined with aerobic exercise to optimize analgesic responses in fibromyalgia: A randomized placebo-controlled clinical trial. Front*.* Hum. Neurosci. 10**,** 68. https://doi.org/10.3389/fnhum.2016.00068.

Mori, F., Codecà, C., Kusayanagi, H., Monteleone, F., Buttari, F., Fiore, S., et al., 2010. Effects of anodal transcranial direct current stimulation on chronic neuropathic pain in patients with multiple sclerosis. J. Pain 11**,** 436-442. https://doi.org/10.1016/j.jpain.2009.08.011.

Morin, A., Léonard, G., Gougeon, V., Cyr, M.P., Waddell, G., Bureau, Y.A., et al., 2017. Efficacy of transcranial direct-current stimulation in women with provoked vestibulodynia. Am. J. Obstet Gynecol. 216**,** 584.e581-584.e511. https://doi.org/10.1016/j.ajog.2017.02.049.

Pegado, R., Silva, L.K., da Silva Dantas, H., Andrade Câmara, H., Andrade Mescouto, K., Silva-Filho, E.M., et al., 2020. Effects of Transcranial direct current stimulation for treatment of primary dysmenorrhea: preliminary results of a randomized sham-controlled trial. Pain Med*.* 21**,** 3615-3623. https://doi.org/10.1093/pm/pnz202.

Samartin-Veiga, N., Pidal-Miranda, M., González-Villar, A.J., Bradley, C., Garcia-Larrea, L., O'Brien, A.T., et al., 2022. Transcranial direct current stimulation of three cortical targets is no more effective than placebo as treatment for fibromyalgia: a double-blind sham-controlled clinical trial. Pain. 163, e850-e861 https://doi.org/10.1097/j.pain.0000000000002493.

Shiasy, Y., Shakiba, S., Taremian, F., Akhavan Hejazi, S.M., and Abasi, A., 2020. The Effectiveness of attention bias modification with and without trans cranial direct current stimulation in chronic low back pain. Iran. J. Psychiatry. 15**,** 112-125.

Soler, D., Moriña, D., Kumru, H., Vidal, J., and Navarro, X., 2021. Transcranial direct current stimulation and visual illusion effect according to sensory phenotypes in patients with spinal cord injury and neuropathic pain. J. Pain 22**,** 86-96. https://doi.org/10.1016/j.jpain.2020.06.004.

Wrigley, P.J., Gustin, S.M., McIndoe, L.N., Chakiath, R.J., Henderson, L.A., and Siddall, P.J., 2013. Longstanding neuropathic pain after spinal cord injury is refractory to transcranial direct current stimulation: A randomized controlled trial. Pain 154**,** 2178-2184. https://doi.org/10.1016/j.pain.2013.06.045.

Yoo, H.B., Ost, J., Joos, W., Van Havenbergh, T., De Ridder, D., and Vanneste, S., 2018. Adding prefrontal transcranial direct current stimulation before occipital nerve stimulation in fibromyalgia. Clin. J. Pain 34**,** 421-427. https://doi.org/10.1097/ajp.0000000000000552.

Young, J., Zoghi, M., Khan, F., and Galea, M.P., 2020. The effect of transcranial direct current stimulation on chronic neuropathic pain in patients with multiple sclerosis: randomized controlled trial. Pain Med. 21**,** 3451-3457. https://doi.org/10.1093/pm/pnaa128.

**Figure legends**

**Figure S5.** Funnel plots with standard errors plotted against effect sizes for determining publication bias in chronic pain studies limited stimulus to the motor cortex at short-term follow-up.

**Figure S6.** Funnel plots with standard errors plotted against effect sizes for determining publication bias in chronic pain intensity at medium-term follow-up.

**Figure S7.** Funnel plots with standard errors plotted against effect sizes for determining publication bias in chronic pain intensity at short-term follow-up.
