# Supplementary material for: Is transcranial direct current stimulation beneficial for treating pain, depression, and anxiety symptoms in patients with chronic pain? A systematic review and meta-analysis
Source: Front Mol Neurosci. 2022 Dec 1;15:1056966. doi: 10.3389/fnmol.2022.1056966 (PMC9752114; doi:10.3389/fnmol.2022.1056966)
Supplement: Supplementary material 4 — Active tDCS compared to Sham tDCS for treating chronic pain with depression or anxiety. [file Data_Sheet_4.docx]

| **Active tDCS compared to Sham tDCS for treating Chronic Pain with Depression or Anxiety** | | | | | | |
| --- | --- | --- | --- | --- | --- | --- |
| **Patient or population:** patients with treating Chronic Pain with Depression or Anxiety **Settings:** laboratory/ clinic **Intervention:** Active tDCS **Comparison:** Sham tDCS | | | | | | |
| **Outcomes** | **Illustrative comparative risks* (95% CI)** | | **Relative effect (95% CI)** | **No of Participants (studies)** | **Quality of the evidence (GRADE)** | **Comments** |
|  | Assumed risk | Corresponding risk |  |  |  |  |
|  | **Sham tDCS** | **Active tDCS** |  |  |  |  |
| **Pain short-term (0 to < 1-week postintervention)** |  | The mean pain short-term (0 to < 1-week postintervention) in the intervention groups was **0.43 standard deviations lower** (0.75 to 0.12 lower) |  | 765 (20 studies) | ⊕⊕⊝⊝ **low**^1^ | SMD -0.43 (-0.75 to -0.12) |
| **Pain middle-term (1 to < 6-week postintervention)** |  | The mean pain middle-term (1 to < 6-week postintervention) in the intervention groups was **0.33 standard deviations lower** (0.71 lower to 0.05 higher) |  | 334 (12 studies) | ⊕⊝⊝⊝ **very low**^2^ | SMD -0.33 (-0.71 to 0.05) |
| **Pain long-term (≥ 6-week postintervention)** |  | The mean pain long-term (≥ 6-week postintervention) in the intervention groups was **0.46 standard deviations higher** (0.35 lower to 1.27 higher) |  | 92 (3 studies) | ⊕⊝⊝⊝ **very low**^3^ | SMD 0.46 (-0.35 to 1.27) |
| **Depression short-term (0 to < 1-week postintervention)** |  | The mean depression short-term (0 to < 1-week postintervention) in the intervention groups was **0.31 standard deviations lower** (0.47 to 0.14 lower) |  | 572 (13 studies) | ⊕⊕⊝⊝ **low**^4^ | SMD -0.31 (-0.47 to -0.14) |
| **Depression middle-term (1 to < 6 weeks postintervention)** |  | The mean depression middle-term (1 to < 6-week postintervention) in the intervention groups was **0.35 standard deviations lower** (0.58 to 0.11 lower) |  | 297 (10 studies) | ⊕⊕⊕⊝ **moderate**^5^ | SMD -0.35 (-0.58 to -0.11) |
| **Depression long-term (≥ 6 weeks postintervention)** |  | The mean depression long-term (≥ 6-week postintervention) in the intervention groups was **0.38 standard deviations lower** (0.64 to 0.13 lower) |  | 245 (6 studies) | ⊕⊕⊕⊝ **moderate**^6^ | SMD -0.38 (-0.64 to -0.13) |
| **Anxiety short-term (0 to 1-week postintervention)** |  | The mean anxiety short-term ( 0 to < 1-week postintervention) in the intervention groups was **0.23 standard deviations lower** (0.47 lower to 0.01 higher) |  | 280 (8 studies) | ⊕⊕⊝⊝ **low**^7^ | SMD -0.23 (-0.47 to 0.01) |
| **Anxiety middle-term (1 to 6 weeks postintervention)** |  | The mean anxiety middle-term (1 to < 6-week postintervention) in the intervention groups was **0.04 standard deviations lower** (0.31 lower to 0.24 higher) |  | 213 (7 studies) | ⊕⊕⊝⊝ **low**^7^ | SMD -0.04 (-0.31 to 0.24) |
| **Anxiety long-term (≥ 6 weeks postintervention)** |  | See comment | Not estimable | 131 (7 studies) | ⊕⊝⊝⊝ **very low**^3^ | SMD -0.26 (-0.51 to -0.02) |
| *The basis for the **assumed risk** (e.g., the median control group risk across studies) is provided in footnotes. The **corresponding risk** (and its 95% confidence interval) is based on the assumed risk in the comparison group and the **relative effect** of the intervention (and its 95% CI). **CI:** Confidence interval; | | | | | | |
| GRADE Working Group grades of evidence **High quality:** Further research is very unlikely to change our confidence in the estimate of effect.  **Moderate quality:** Further research is likely to have an important impact on our confidence in the estimate of effect and may change the estimate. **Low quality:** Further research is very likely to have an important impact on our confidence in the estimate of effect and is likely to change the estimate. **Very low quality:** We are very uncertain about the estimate. | | | | | | |
| ^1^ Downgraded once for study limitations due to high or unclear risk of bias and once for inconsistency due to heterogeneity. ^2^ Downgraded once for study limitations due to high or unclear risk of bias and once for inconsistency due to heterogeneity and once for the asymmetrical distribution of the results in the funnel plot. ^3^ Downgraded once for study limitations due to high or unclear risk of bias, once for imprecision due to low participant numbers, and once for the number of comparisons was lower than 10 and it was not possible to exclude the presence of a publication bias. ^4^ Downgraded once for study limitations due to high or unclear risk of bias and once for the asymmetrical distribution of the results in the funnel plot. ^5^ Downgraded once for study limitations due to high or unclear risk of bias ^6^ Downgraded once for the number of comparisons was lower than 10 and it was not possible to exclude the presence of a publication bias.  ^7^ Downgraded once for study limitations due to high or unclear risk of bias and once for the number of comparisons was lower than 10 and it was not possible to exclude the presence of a publication bias. | | | | | | |
