# Supplementary material for: Is transcranial direct current stimulation beneficial for treating pain, depression, and anxiety symptoms in patients with chronic pain? A systematic review and meta-analysis
Source: Front Mol Neurosci. 2022 Dec 1;15:1056966. doi: 10.3389/fnmol.2022.1056966 (PMC9752114; doi:10.3389/fnmol.2022.1056966)
Supplement: Supplementary material 5 — Abbreviations. [file Data_Sheet_5.docx]

| Supplementary material 5-Abbreviations | |
| --- | --- |
| tDCS | Transcranial direct current stimulation |
| NIBS | Non-invasive brain stimulation |
| RCT | Randomized controlled trial |
| ES | Effect size |
| SES | Summary effect size |
| CI | Confidence interval |
| SD | Standard deviation |
| SE | Standard error |
| IQR | Interquartile range |
| SMD | Standard mean difference |
| PFC | Prefrontal cortex |
| DLPFC | Dorsolateral prefrontal cortex |
| M1 | Motor cortex |
| dACC | Dorsal anterior cingulate cortex |
| OIC | Operculo-insular cortex |
| VAS | Visual analogue scale |
| VNS | Visual Numeric Scale |
| NRS | Numerical rating scale |
| DVPRS | Defense and Veterans Pain Rating Scale |
| HADS | Hospital Anxiety and Depression Scale |
| BDI | Beck Depression Inventory |
| HAMD | Hamilton Depression Scale |
| PHQ-9 | Patient Health Questionnaire-9 |
| HAMA | Hamilton Anxiety Scale |
| PASS-20 | Pain Anxiety Symptoms Scale |
| HPA | Hypothalamic pituitary adrenocortical |
| CLBP | Chronic low back pain |
| CNLBP | Chronic non-specific low back pain |
| AE | Aerobic exercise |
| ABM | Attention bias modification |
| VI | Visual illusion |
| MT | Mirror therapy |
| PAG | Periaqueductal gray |
| rTMS | Repetitive transcranial magnetic stimulation |
